# Variant-Specific Interactions at the Plasma Membrane: Heparan Sulfate’s Impact on SARS-CoV-2 Binding Kinetics

**DOI:** 10.1021/acs.analchem.4c04283

**Published:** 2025-02-20

**Authors:** Dario Valter Conca, Fouzia Bano, Małgorzata Graul, Julius von Wirén, Lauriane Scherrer, Hudson Pace, Himanshu Sharma, Justas Svirelis, Konrad Thorsteinsson, Andreas Dahlin, Marta Bally

**Affiliations:** 1 Department of Clinical Microbiology, Umeå University, Umeå 901 87, Sweden; 2 Wallenberg Centre for Molecular Medicine (WCMM), Umeå University, Umeå 901 87, Sweden; 3 Umeå Centre for Microbial Research (UCMR), Umeå University, Umeå 901 87, Sweden; 4 Department of Medical Biochemistry and Biophysics, Umeå University, Umeå 901 87, Sweden; 5 Laboratory for Molecular Infection Medicine Sweden (MIMS), Umeå University, Umeå 901 87, Sweden; 6 Department of Chemistry and Chemical Engineering, Chalmers University of Technology, Gothenburg 412 96, Sweden

## Abstract

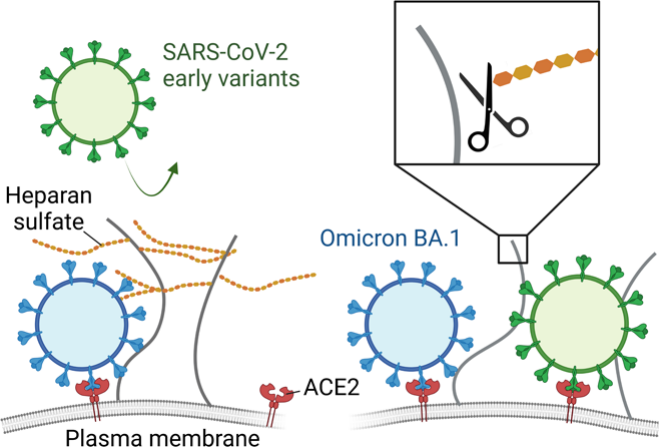

The spread of SARS-CoV-2 led to the emergence of several variants of concern (VOCs). The spike glycoprotein, responsible for engaging the viral receptor, exhibits the highest density of mutations, suggesting an ongoing evolution to optimize viral entry. This study characterizes the bond formed by virion mimics carrying the SARS-CoV-2 spike protein and the plasma membrane of host cells in the early stages of virus entry. Contrary to the traditional analysis of isolated ligand–receptor pairs, we utilized well-defined biomimetic models and biochemical and biophysical techniques to characterize the multivalent interaction of VOCs with the complex cell membrane. We observed an overall increase in the binding affinity for newer VOCs. By progressively reducing the system complexity, we identify heparan sulfate (HS) as a main driver of this variation, with a 10-fold increase in affinity for Omicron BA.1 over that of the original strain. These results demonstrate the essential role of coreceptors, particularly HS, in the modulation of SARS-CoV-2 infection and highlight the importance of multiscale biophysical and biochemical assays that account for membrane complexity to fully characterize and understand the role of molecular components and their synergy in viral attachment and entry.

## Introduction

Angiotensin converting enzyme 2 (ACE2) was identified as the entry receptor for severe acute respiratory syndrome coronavirus 2 (SARS-CoV-2) only weeks after the isolation of the virus,^1^ and since then, its interaction with the viral glycoprotein spike has been thoroughly characterized.^2^ However, the isolated interaction with the viral receptors is a great simplification of the reality of the first interaction between the virus and the complex host membrane environment, containing hundreds of molecule types.^3^ A viral particle approaching the cell must navigate through plasma membrane components before binding the viral receptor.^4^ These important interactions and their interplay are often overlooked due to the challenges arising from studying and interpreting such a complex environment. Investigations of the SARS-CoV-2 entry process are further complicated by the fact that the virus mutates quickly,^5^ with the highest mutation rates observed in the receptor-binding domain (RBD) of spike. This suggests an optimization of the interaction with the cell surface, correlating with higher infectivity reported for emerging variants of concern (VOCs).^6^ Omicron (BA.1), in particular, was characterized by a rapid increase in the number of mutations, which was accompanied by a shift in viral tropism toward the upper respiratory tract and generally milder symptoms.^7^ Despite these changes, the strength of the interaction between spike and ACE2 remained fairly constant among VOCs with affinities reported in the 0.5–50 nM range.^6,8^ This suggests an additional contribution of attachment factors, whose role remains elusive.

Several attachment factors have been proposed for SARS-CoV-2,^9^ including heparan sulfate (HS) proteoglycans.^10,11^ HS is a heterogeneously sulfated polysaccharide, with varying sulfation patterns, which is widely expressed as a component of the glycocalyx at the cell surface of all animal tissues.^12^ It is involved in the initial binding of numerous viruses where the particles often form weak multivalent interactions prior to the engagement of the main entry receptor.^13^ A significant increase in the affinity to HS was observed for Omicron, with an ∼4-fold increase as compared to the original Wuhan strain, possibly driven by the increased overall positive charge of spike interacting with the negatively charged HS.^14^ This increase has been hypothesized to explain the virus tropism shift.^15^ However, the role and importance of HS in the entry process remain to be elucidated as studies have primarily focused on isolated spike–HS pairs rather than considering the molecular complexity of the membrane environment. This makes it challenging to discern the role of multivalency, avidity, and the relative importance of distinct surface receptors in the overall virus interaction at the membrane.

The direct study of the molecular interaction between SARS-CoV-2 and the whole plasma membrane has been hindered by the need for stable and biologically significant models for both the viral particles and the host cell surface. On the particle side, Staufer et al. recently proposed the use of soluble spike protein immobilized onto synthetic liposomes, which resemble SARS-CoV-2 virions in size and shape,^16^ as a particle model to study virus binding. This system, while highly simplified and not suitable for infection assays, allows for better control of the sample purity, uniformity, and composition, compared to inactivated virus or virus-like particles. On the membrane side, the determination of the kinetic parameters of molecular bonds requires the observations of single-particle attachment and detachment at equilibrium^17,18^ under stable experimental conditions. Live cells are not suitable since internal cellular processes and their response to stimulation may interfere with the membrane composition and characteristics. Fixing cells can cause artificial biomolecule clustering^19^ and prevents the mobility of membrane components, making biologically relevant receptor rearrangements during the interaction with the virion impossible. An alternative is reconstituting the cellular plasma membrane on a glass surface^20^ where microscopy observation with single-particle resolution can be performed via total internal reflection fluorescent microscopy (TIRFM).^18,21^ This is achieved using native supported lipid bilayers (nSLBs) obtained from the purified plasma membrane of mechanically disrupted cells.^22^ While requiring dilution of the native material with synthetic PEGylated lipids for successful bilayer formation, nSLBs preserve the native membrane composition as well as the mobility of membrane components.^23^ These biomimetic models allow unprecedented control over the experimental setup and thus precise determination of the kinetic parameters of biological interaction both at the single-particle and single-molecule level.

In this work, we use well-characterized and controlled biomimetic systems to perform a multiscale biophysical study elucidating the effects of avidity on the interaction between SARS-CoV-2 VOCs and the plasma membrane of physiologically relevant lung epithelial cells, the role of HS in modulating the interaction, and how it changes across VOCs.

## Experimental Section

### Small Unilamellar Vesicle (SUV) Production

1-Palmitoyl-2-oleoyl-glycero-3-phosphocholine (POPC, 850457P), 1,2-dioleoyl-*sn*-glycero-3-[(*N*-(5-amino-1-carboxypentyl) iminodiacetic acid) succinyl] (DGS-NTA, 790528P), 1,2-dioleoyl-*sn*-glycero-3-phosphoethanolamine-*N*-(lissamine rhodamine B sulfonyl) (Liss Rhod, 810150C), 1,2-dioleoyl-*sn*-glycero-3-phosphoethanolamine-*N*-(capbiotinyl) (bioDOPE, 870273), and *N*-palmitoyl-sphingosine-1-{succinyl[methoxy(polyethylene glycol)5000]} (PEG-cer, 880280P) were purchased from Avanti Polar Lipids (Alabaster, AL, USA) and Oregon Green 488. 1,2-Dihexadecanoyl-*sn*-glycero-3-phosphoethanolamine (OG-DHPE, O12650) was from Thermo Fisher Scientific (Waltham, MA, USA). SUVs were produced using the established lipid film hydration and extrusion method further detailed in the Supporting Information.

### Glycosaminoglycans and Proteins

Heparan sulfate purified from porcine mucosa (HS; GAG-HS01 BN1) was purchased from Iduron (UK). Lyophilized end-biotinylated HA (b-HA) was kindly provided by Innovent e.V., Technologieentwicklung (Jena, Germany). All lyophilized HS samples were dissolved by gently mixing overnight at 4 °C in Milli-Q (Millipore integral system, Molsheim, France) and biotinylated at their reducing end by oxime oxidation as described in ref (24). Soluble SARS-CoV-2 VOCs spike stabilized to retain the prefusion conformation with 10× His-tag at the C-terminus of each monomer were purchased from ACROBiosystems (Newark, DE, USA) (product numbers: Wuhan, SPN-C52H9; Alpha, B.1.1.7, SPN-C52H6; Delta, B.1.617.2, SPN-C52He; Omicron, BA.1, SPN-C52Hz). The spikes were resuspended at 0.66 mg/mL in Milli-Q and 0.1% BSA, flash-frozen, and stored at −80 °C. Streptavidin was obtained from Sigma-Aldrich (Burlington, MA, USA).

### Spike Functionalization of NTA-Liposomes

On the day of the experiment, POPC:DGS-NTA:Liss Rhod vesicles were diluted in HBS (150 mM NaCl and 10 mM Hepes, pH 7.4) to a concentration of 100 μg/mL. NiCl_2_ was added to a final concentration of 20 mM for a final volume of 100 μL, and the vesicles were incubated in the dark at room temperature for 30 min. NiCl_2_ was removed using MicroSpin S-200 HR columns (27512001, Cytiva, Sweden) as by the manufacturer's instructions. For spike immobilization, soluble spike was added to activated vesicles to a final concentration of 60 μg/mL. After mixing, the sample was incubated at room temperature in the dark for 2 h. Unbound spike was removed with 20 μL of prewashed Capto Core 700 beads (17548102, Cytiva) in PBS and incubated with the liposomes for 30 min at 4 °C under gentle rotation. The solution was then centrifuged at 1000*g* for 1 min to separate the beads from the vesicles, and the supernatant was run through MicroSpin S-400 HR columns (27514001, Cytiva) using the same protocol as for MicroSpin S-200 HR columns with a centrifugation speed of 600*g*. The elute containing spike-decorated liposomes was collected and stored at 4 °C until use the same day. Spike aliquots were kept at 4 °C for a maximum of 3 days.

### Spike Quantification via Western Blotting

The amount of spike captured by NTA-liposomes was quantified via Western blotting as described in the Supporting Information. The spike content for each variant was estimated by measuring the band intensities at a MW of ∼210 kDa using an existing plugin in ImageJ and using a calibration curve (Figure S1) obtained by the serial dilution (3×) of soluble spike (Delta).

### Spike-Decorated Liposome Imaging Using Cryogenic Electron Microscopy (Cryo-EM)

Freshly functionalized liposomes were plunge-frozen in liquid ethane and imaged using a Glacios Electron microscope (Thermo Scientific). See the Supporting Information for details.

### Cell Binding Assay

Calu-3 cells from ATCC (HTB55, Manassas, VA, USA) were cultured in high-glucose DMEM (D5648, Sigma-Aldrich), 10–20% fetal bovine serum (FBS, SV30160.03, HyClone, USA), and 1% penicillin/streptomycin (PenStrep, 10,000 units/mL penicillin and 10,000 μg/mL streptomycin, 15140-122, Gibco). Four days prior to the experiment, cells were seeded on a glass-bottom 96-well plate at a density of ∼10^5^ cells/well. On the day of the experiment, cells were washed in PBS and incubated on ice with 10 μL of spike-decorated liposome (1% molar concentration of DNG-NTA lipids) solution in 40 μL of DMEM + 3% FBS for 1 h. They were then thoroughly washed in ice-cold PBS and fixed in ice-cold 4% paraformaldehyde (PFA) for 10 min. The wells were rinsed in PBS and kept in PBS at 4 °C until imaging on a Nikon Ti2-E microscope in a widefield configuration, using a Spectra III solid-state light source (555 nm emission wavelength), a multiband pass filter cube 86012v2 DAPI/FITC/TxRed/Cy5 (Nikon Corporation, Melville, USA), a CFI Apochromat TIRF 60XC oil-immersion 60× objective (Nikon, NA: 1.49), and a Prime 95B sCMOS camera (Teledyne Photometrics, Birmingham, UK). A *z*-stack was acquired for 5 positions per well using an axial step size of 0.3 μm and a range of 25 μm. The fluorescent particles attached to cells were counted by performing a maximum intensity projection on the *z*-stack and then applying an in-house 2D peak-finder algorithm (see the kinetic analysis section) to detect all of the intensity peaks with prominence higher than 50. The result was normalized by the particle concentration in each sample, measured using “bouncing particle analysis” (BPA) as described previously.^25^ Additional details are presented in the Supporting Information and Figure S2.

### Production of Native Supported Lipid Bilayers (nSLB), HS-Functionalized Synthetic Bilayers and HS Enzymatic Removal

Native membrane vesicles and nSLBs (12.5% of the cell material by surface area) from Calu-3 cells were produced and characterized as described in refs (18) and (22). The presence of ACE2 in native membrane vesicles was demonstrated by Western blotting (Figure S6). The lipids embedded nSLBs showed a high mobile fraction (97% ± 1) and a diffusion coefficient of 2.33 ± 0.09 μm^2^/s (Figure S3), indicating high-quality bilayers. See the Supporting Information for further details. Heparan sulfate was immobilized on a glass surface using a biotinylated supported lipid bilayer (POPC:bioDOPE 95:5 molar ratio) through a streptavidin bridge.^17^ Enzymatic removal of HS from Calu-3 nSLBs was achieved with a cocktail of heparinase I and III (H2519 and H8891, Sigma-Aldrich) incubating 2 units/mL in 20 mM Tris, 100 mM NaCl, and 1.5 mM CaCl_2_ at pH 7.0 for 1 h. Almost complete removal of HS was verified by immunostaining with anti-HS antibodies and flow cytometry or microscopy analysis, as described in the Supporting Information (Figures S4 and S5).

### Imaging and Analysis for Equilibrium Fluctuation Analysis of Spike-decorated Liposomes

Spike-decorated liposome (1 mol % DNG-NTA lipids) attachment and detachment to SLBs were imaged by acquiring time lapses with a Nikon Ti2-E microscope (see the Cell Binding Assay) in TIRF mode using a 561 nm excitation laser. After the bilayer formation and functionalization, 5 μL of fluorescent spike-decorated liposomes was added to 5 μL of PBS in each well and incubated for at least 1 h to reach kinetic equilibrium, i.e., the average number of bound and free particles does not vary with time. Images (704 × 704 pixels, with a resolution of 0.183 μm/pixel) were acquired for 1.5–3 h, in all wells simultaneously and at 4 random positions per well. The frame rate was set to 25–40 s per frame and the exposure time to 100 ms.

Image registration was performed with an in-house MATLAB script to remove subtle movements due to the parallel acquisition of several positions.^21,25^ Particles were then tracked in the stabilized movies as described previously,^25^ using an in-house MATLAB script. The particle concentration was measured by BPA, and the average concentration was 6.1 ± 3.5 × 10^8^ particles/mL.

The traces were analyzed for the determination of the kinetic parameters of the interaction using equilibrium fluctuation analysis (EFA).^21^ Under kinetic equilibrium conditions, the cumulative attachment rate is expected to be linear. Accordingly, the relative variation of the association rate constant *k*_on_ was determined by fitting the cumulative arrival time of the particle on the surface to *y* = *r*_a_*t* + *y*_0_, where *r*_a_ is the particle arrival rate and *y*_0_ considers the particles already bound at the beginning of the acquisition (*t* = 0) (Figure S11A). Once normalized by the particle concentration in solution, the measured arrival rate, *k*_m_, is proportional to *k*_on_ and can be used to compare the attachment rates between samples. The dissociation rate constat *k*_off_ was calculated by fitting the number of attached particles as a function of the residence time using *y* = *A* exp(− *k*_off_*t*) + *C*. This approach implies that the detachment rate of the multivalent interaction established by the particles can be approximated to a single rate constant as documented in the literature^26^ and confirmed by the good agreement with our experimental results (Figure S11B). Only the particles landing in the first half of the movie were considered to avoid bias toward short residence times.

All kinetic parameters are reported as normalized to the value obtained for Omicron for each experiment. In this way, we accounted for the experimental variations due to changes in the liposome and bilayer concentration and composition between experiments. Non-normalized data can be found in Tables S1–S3.

### Atomic Force microscopy-Based Single Molecule Force Spectroscopy (AFM-Based SMFS) Experiments

The atomic force microscopy (AFM) tip was functionalized with a PEG linker following the protocol in refs (27) and (28) and described in detail in the Supporting Information. HS-functionalized bilayers were formed as described above on a cleaned glass coverslip and fixed on a stainless-steel AFM holder using a bicomponent Twinsil glue (Picodent, Germany). This created a well that held a maximum volume of about 200 μL.

All SMFS experiments were performed in PBS at room temperature using a JPK/Bruker 4XP BioScience with JPK SPM Control Software v.7 (JPK/Bruker, Berlin, Germany). Force–distance (FD) curves were recorded using the cantilevers with nominal spring constants of 10 or 30 pN/nm of the MSCT probes. All tips used had a spring constant within 10% of the specification as measured using the thermal noise method.^29^ The spike-functionalized tip was moved toward the HS surface (ramp size = 400 nm) at a speed of 1 μm/s until it was in contact with the surface (force load threshold of 600 pN). The tip was left to interact with the surface (dwell time) and then retracted. For dissociation rate constant () measurements, retract speeds were varied from 1 to 7 μm/s, and 1500–2500 FD curves were recorded by keeping the approach speed fixed to 1 μm/s and no dwell time. For association rate constant () measurements, both approach and retraction speeds were set to 1 μm/s to probe the probability of bond formation (binding probability, BP) as a function of dwell time. For this, 400–500 FD curves were recorded for dwell times of 50, 100, 150, 200, 300, 500, and 1000 ms. For each condition, at least 2 experiments with independently prepared tips and surfaces were performed.

### SMFS Data Analysis

All SMFS data were processed using JPK data processing software (JPK/Bruker, Germany). The retract curves were analyzed using the freely joint chain (FJC) model keeping both contour and Kuhn lengths as fitting parameters.^30^ The curves with unbinding events occurring at less than 10 nm from the surface were discarded to exclude nonspecific interactions. The binding probability was calculated as the fraction of curves displaying rupture events. From the FJC fitting, the instantaneous loading rate (*r*) was determined by multiplying the effective spring constant, i.e., the slope of the FJC fit close to the rupture point, by the retract speed. Force histograms were constructed to calculate the mean rupture force from every unbinding event at the same retraction speed or loading rate and fitted with a Gaussian function. Next, the most probable rupture force (*F*) was plotted as a function of *r* to create dynamic force spectra (DFS) plots. DFS data were then fitted with the Bell–Evan model () to extract the intrinsic bond kinetic parameters (i.e.,  and *x*_β_) using linear regression analysis, where *x*_β_ is the width of the energy barrier,  is the rate constant at *F* = 0, and *k*_B_*T* is the product of the Boltzmann constant and the temperature.^31^

To determine , the binding probability as a function of dwell time, *t*, was fitted with the following pseudo-first-order kinetic function: , where *A* is the maximum measured BP, *t*_0_ is the lag time, and τ is the interaction time between spike and HS.  was determined from the following function: , where *C*_eff_ is the effective concentration , where *r*_eff_ is the radius of the interaction sphere, assumed to be equal to the equilibrium length of the PEG linker, ∼2 nm, plus the size of spike, ∼20 nm, *n*_b_ is the number of potential bonds formed within the hemisphere in which interaction can take place (1 in our case, as majority of FD curves show only a single unbinding event), and *N*_A_ is Avogadro’s number.^32^ The single-molecule dissociation constant was calculated as . The standard deviation of the kinetic constants was determined by propagating the experimental and fitting errors through the above-mentioned formulas.

## Results

### Spike-Decorated Liposomes Are Suitable Virion Mimics

**Figure 1:**
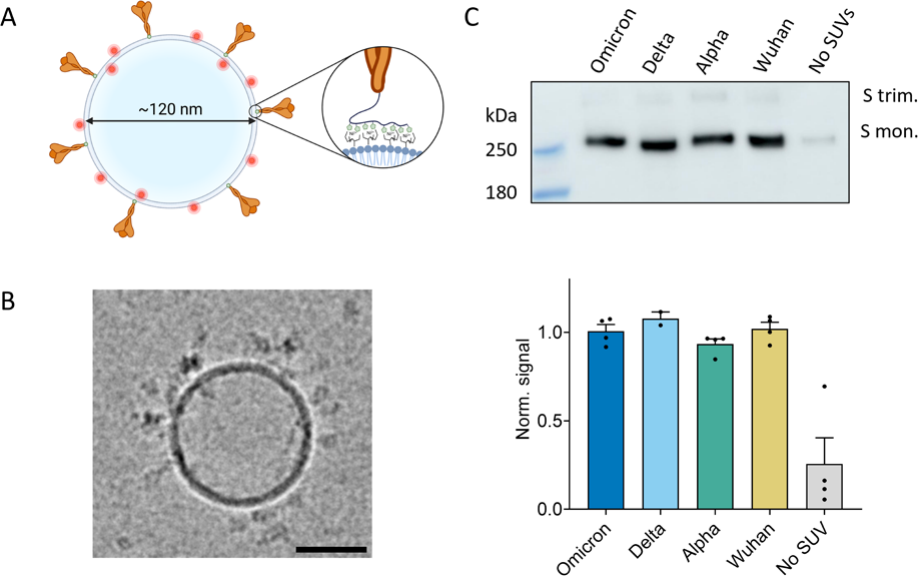
Spike-decorated liposomes are suitable SARS-CoV-2 virion mimics for single-particle studies. (A) Schematic of a spike-decorated liposome. A soluble spike, in orange, is attached to the liposomes via interaction between the NTA-conjugated lipids and the poly-His-tag on the C-terminus of the protein. (B) Cryo-EM image of a spike-decorated liposome showing spike (Alpha) proteins bound to the surface and correctly oriented. Scale bar: 50 nm. (C) Top: example of a Western blot of lysed spike-decorated liposomes immunostained with an anti-His-tag antibody. The last lane to the right (no SUVs) shows the soluble spike remaining after filtration via Capto Core beads in the absence of NTA-liposomes. Bottom: bar plot showing the relative spike loading obtained from Western blotting of spike-decorated liposomes from 4 independent repeats (2 for Delta). Signal is normalized by the average of all variants for each repeat.

We implemented fluorescent spike-decorated liposomes as virion mimics to study the role of multivalency in spike attachment to the cell membrane. His-tagged soluble spike trimers were immobilized on POPC liposomes containing rhodamine-conjugated lipids and DGS-NTA lipids (Figure 1A). The average particle diameter was ∼125 nm, increasing to ∼145 nm after spike immobilization (Figure S7) comparable to the size of the SARS-CoV-2 virion.^33^ This procedure resulted in the production of uniform, pure, and bright particles, easily tracked using TIRFM. Spike was found to be stably immobilized to the liposome bilayer, as further verified in quartz crystal microbalance with dissipation monitoring (QCM-D) experiments and immunostaining of liposomes with anti-SARS-CoV-2 antibodies (Figures S8A and S10). The specificity of the bond was confirmed by the near complete release of spike using imidazole, a Ni-NTA binding competitor (Figure S8A), and blocking of the spike-NTA interaction by anti-His-tag antibodies (Figure S8B). Cryo-EM images of the liposomes confirmed that spike trimers are oriented as on SARS-CoV-2 virions (Figure 1B and Figure S9). Protein loading was found to be independent of the spike variant, as confirmed by Western blot analysis and QCM-D in Figure 1C and Figure S8B, respectively.

We subsequently explored the possibility of controlling the spike concentration on the particle surface by adjusting the NTA content. Relative spike capture was quantified through Western blot analysis (Figure S8E). The spike concentration in the sample depends linearly on the percentage of NTA in the SUVs and plateaus at concentrations higher than 1%. This was also confirmed using QCM-D (Figure S8C). In addition, using surface plasmon resonance and quantitative Western blot evaluation of the spike content in liposome lysates, we determined that the spike density resembles that observed on SARS-CoV-2 virions (∼50 proteins/particle, Supporting Information, Figures S1 and S8D).^33,34^ Spike-decorated liposomes not only allow for a direct comparison between the spike of SARS-CoV-2 variants but also closely resemble SARS-CoV-2 virions in size and spike distribution, density, orientation, and functionality. For these reasons, they are an attractive model to the study of virus–membrane interaction over commonly used alternatives such as pseudotypes or inactivated viruses.

### Increased Binding to Pulmonary Cells for Omicron

**Figure 2:**
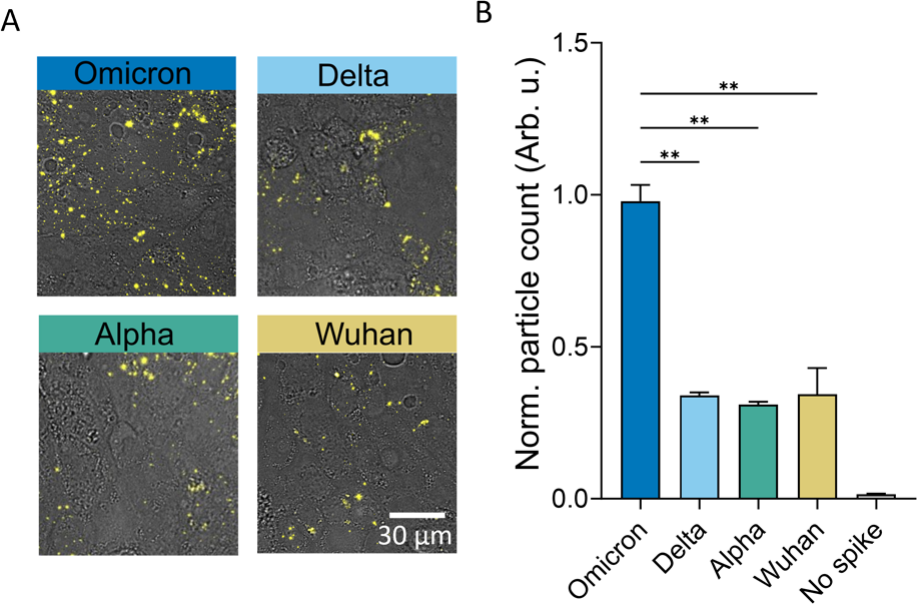
Omicron efficiently binds to Calu-3 cells. (A) Representative images of spike-decorated liposomes, in yellow, bound to Calu-3 cells, for all VOCs used in the study. (B) Relative quantification of the number of particles bound, adjusted by the vesicle concentration. Data were calculated from the average of 5 replicates from two independent experiments. Statistical significance was calculated using the Brown–Forsythe and Welch ANOVA test. **p < 0.01. All VOCs show a *p* < 0.001 significantly increased binding with respect to SUV without spike (no spike).

We tested the ability of spike-decorated liposomes to specifically bind to Calu-3 pulmonary epithelial cells. Calu-3 are derived from the lower respiratory tract, one of the main targets of the virus, and display an entry and replication tropism similar to that observed in air–water interface cultures and *in vivo*.^35^ Cells were incubated on ice with spike-decorated liposomes and fixed prior to epifluorescence imaging (Figure 2A). By counting the bound particles, we observed a significant ∼2- to 3-fold increase in binding for Omicron compared to all the previous VOCs (Figure 2B). No significant differences were observed between Alpha, Delta, and Wuhan strains. Further, particles lacking spike showed negligible binding, excluding electrostatic effects due to the Ni-NTA positive charge. We conclude that Omicron displays a significant increase in attachment to Calu-3 cells as compared to previous VOCs.

### Faster Association Drives Improved Multivalent Affinity for Omicron

**Figure 3:**
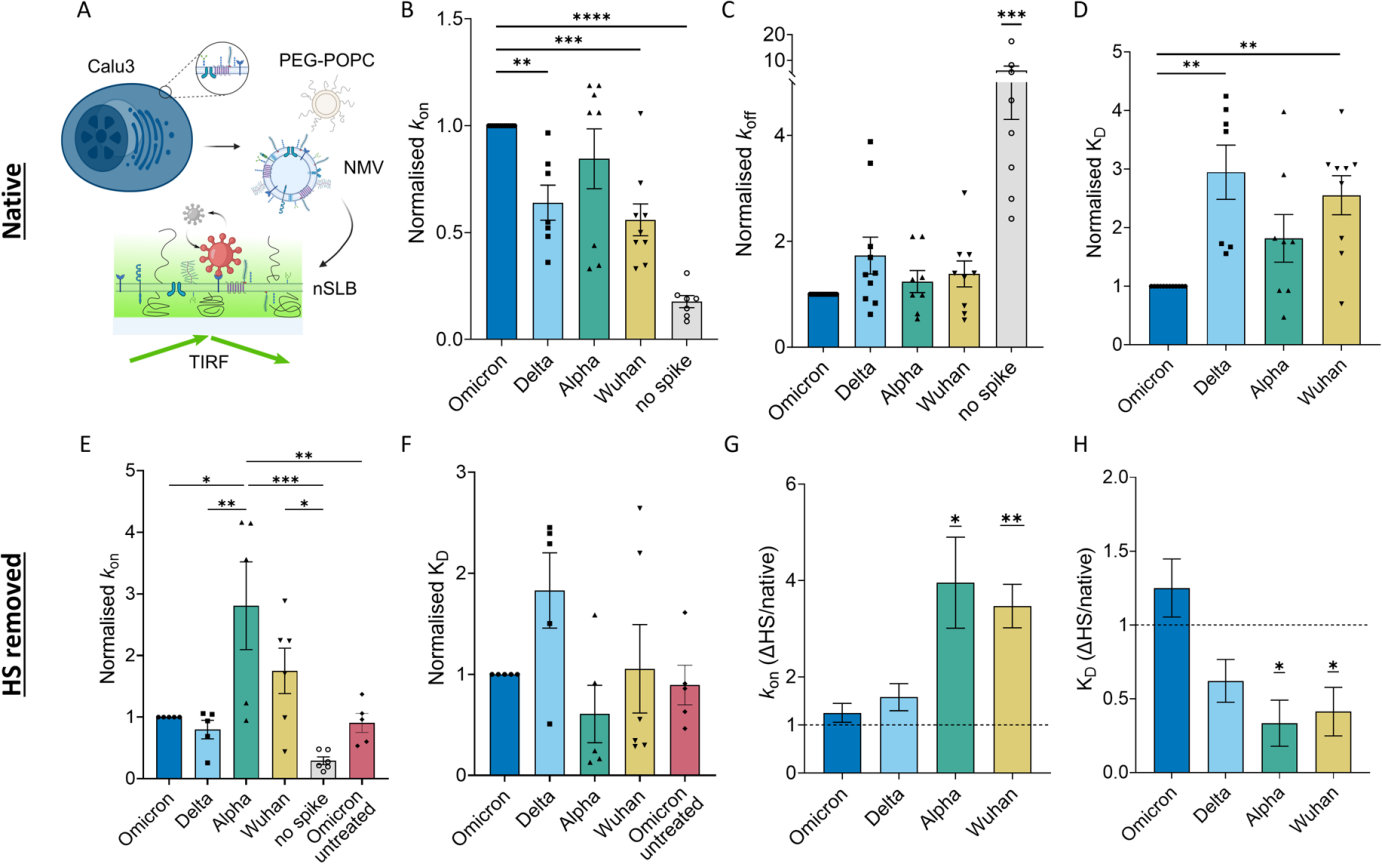
Omicron shows increased multivalent affinity only in the presence of HS. (A) Sketch showing the formation of a native supported lipid bilayer (nSLB) from Calu-3-derived native membrane vesicles (NMVs) and the TIRFM-based EFA experimental setup (see the Experimental Section). (B–D) Kinetic parameters (*k*_on_, *k*_off_, and *K*_D_ in B, C, and D, respectively) measured via EFA of the interaction between spike-decorated vesicles and Calu-3-derived nSLBs. (E, F) Normalized kinetic parameters (*k*_on_ in E and *K*_D_ in F) measured for each variant after the treatment with the heparinase I+III cocktail and Omicron on an nSLB mock-treated with a digestion buffer. Values are normalized in each experiment to Omicron. A summary of the data before normalization is presented in Table S2. (G, H) Fold-change of the *k*_on_ (G) and *K*_D_ (H) due to heparan sulfate removal (ΔHS). Statistical significance was calculated using one-way ANOVA tests in B–F and one-sample *t* tests (hypothetical value = 1) in G and H. *p < 0.05, **p < 0.01, ***p < 0.001, and ****p < 0.0001. In C, *** indicates the lowest significance between the negative control and all the other conditions tested.

Building on the qualitative indications of the cell binding assay, we further characterized particle attachment to the plasma membrane by investigating the binding kinetics of spike-decorated liposomes onto nSLBs derived from Calu-3 cells. These were formed by fusing PEGylated POPC vesicles and the native membrane material. After bilayer formation, we incubated spike-decorated liposomes until the system reached equilibrium, i.e., a constant number of particles was bound to the surface. We then performed TIRF-based EFA by observing single spike-decorated liposome attachment and detachment from the surface at kinetic equilibrium,^26^ as schematically shown in Figure 3A. Detection and tracking of individual particles from the time lapses allowed us to extract the kinetic parameters of the multivalent interaction between the spike-decorated vesicles and the nSLB. In particular, we determined the dissociation rate constant (*k*_off_) and measured the attachment rate, as shown in Figure S11. The latter, once adjusted for the particle concentration in solution, yields the measured association constant, *k*_m_, which in turn, is proportional to the association rate constant (*k*_on_). The results for all VOCs are shown in Figure 3B,C. We report a negligible attachment in the absence of spike, confirming that the system has a low nonspecific signal. Most interestingly, we observed a significant increase in the association rate of Omicron, compared to all the other VOCs tested, except for Alpha. No significant difference between VOCs was observed for *k*_off_, although Omicron displayed the slowest dissociation rate on average (Figure 3C) and an increased fraction of particles that remain irreversibly linked to the substrate throughout the experiment (irreversible fraction) (Figure S12A). Similar dissociation rates may be due to the high-affinity interaction with the ACE2 receptor for all VOCs.^6,10^ Indeed, we speculate that after the initial attachment, possibly through several attachment factors, the particle creates a stable bond with ACE2, which dominates the detachment kinetics. In addition, the creation of multiple bonds to the membrane, which stabilizes the interaction and strongly reduces detachment (the irreversible fraction is ∼50% for all VOCs; see Table S1), might mask differences between VOCs in the detachment kinetics with possible coreceptors. Combining association and dissociation rates yields a relative dissociation constant (*K*_D_^*^ = *k*_off_/*k*_m_), i.e., the relative difference in multivalent affinity of the system between VOCs; we observed an ∼3-fold reduction in *K*_D_^*^ (i.e., higher affinity) for Omicron when compared to Wuhan and Delta, with a trend toward an advantage over Alpha as well, although without statistical significance (Figure 3D). This is supported by a statistically significant higher surface coverage on the nSLB for Omicron than all the other vairiants, including Alpha (Figure S12B).

### Heparan Sulfate Reduces Binding to the Plasma Membrane for All VOCs but Omicron

We aimed to identify the main factor responsible for the increase in the interaction of Omicron with the plasma membrane. Since HS has been shown to interact with spike and is believed to be an important attachment factor for SARS-CoV-2,^10,36^ we proceeded with enzymatically removing HS from the nSLB surface using heparinase I and III. As in the untreated case, we observed the main differences in the arrival rate with a significantly higher *k*_m_ for the Alpha variant compared to all the other tested VOCs but Wuhan. Omicron, which had the highest attachment rate on untreated nSLBs (Figure 3B), now showed a lower association than Wuhan and Alpha (2- and 3-fold reduction, respectively) and a similar level to Delta (Figure 3E). This was also reflected in a trend toward higher *K*_D_^*^ than Alpha (Figure 3F) and lower surface coverage (Figure S12E). As for the untreated nSLBs, no clear differences were observed in particle dissociation, except for particles lacking spikes (Figure S12C). Similar irreversible fractions were also measured for all VOCs, as shown in Figure S12D. Comparing the association rate before and after the enzymatic treatment of the nSLB (Figure 3G and Figure S12F) reveals that the Omicron binding was not significantly affected by HS removal. Conversely, for Alpha and Wuhan, the attachment rate was significantly increased >3-fold, resulting in a >60% decreased *K*_D_ (Figure 3H and Figure S12G). Delta also displayed a similar trend, with an ∼40% increase in *k*_on_ and ∼30% reduction of *K*_D_ after heparinase treatment, albeit this was not statistically significant. Altogether, these results indicate that HS reduces SARS-CoV-2 binding to the plasma membrane for all VOCs but Omicron, presumably by limiting access to ACE2 or other surface receptors.

### Omicron Uses Heparan Sulfate as a High-Affinity Attachment Factor

**Figure 4:**
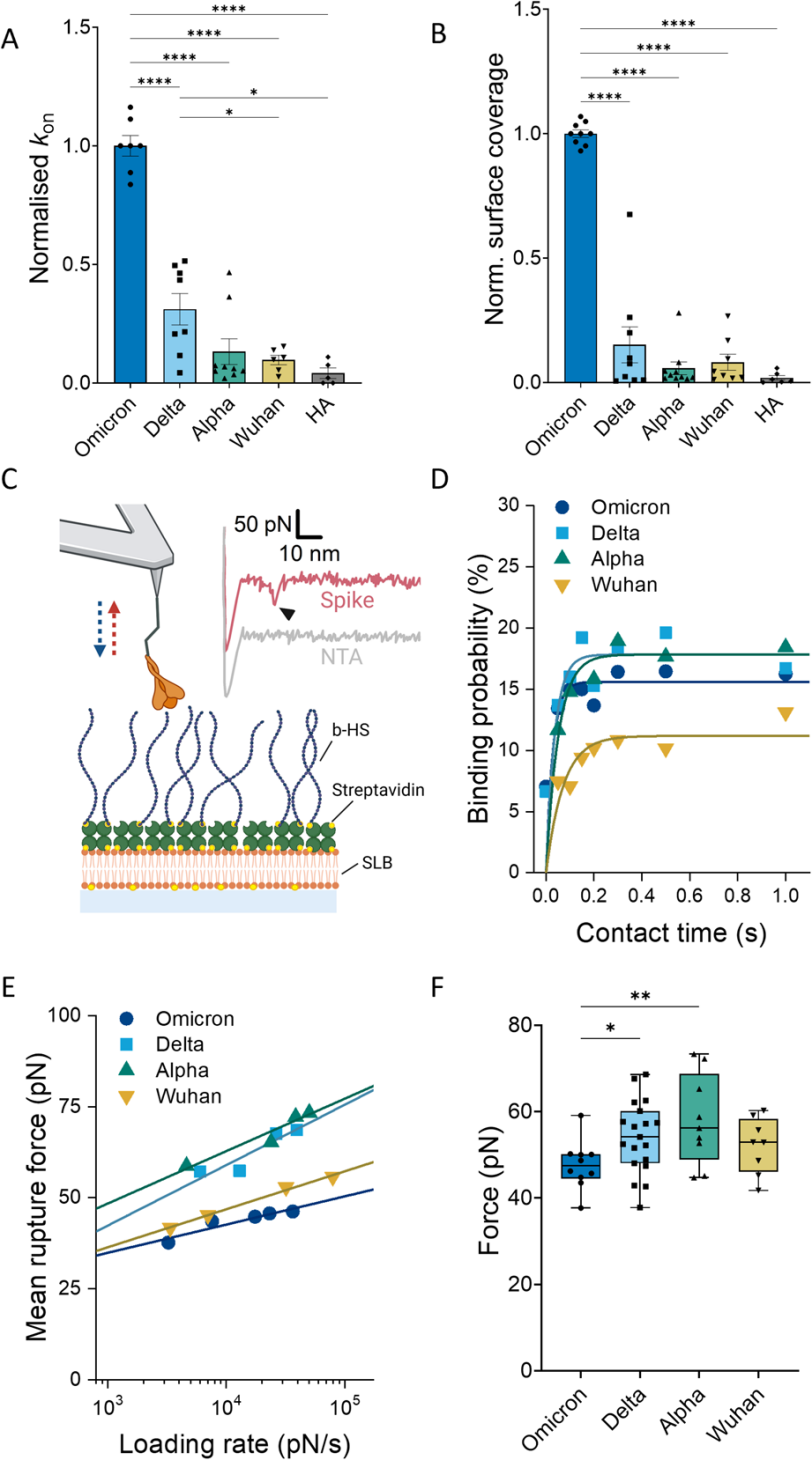
Large variations in HS binding kinetics between VOCs. (A) Association rate constant for spike-decorated liposomes binding to surface-immobilized HS. HA is used instead of HS as a negative control with liposomes decorated with Omicron spike. (B) Average number of particles bound to the HS surface at kinetic equilibrium. The values in A and B are normalized to the average of Omicron for each experiment. (C) Schematic representation of the AFM-based SMFS setup used to quantify the binding interaction between spike VOCs and biotinylated HS (b-HS) immobilized on an SLB via a streptavidin bridge. Inset: representative FD curves with (red) and without (gray) spike. The arrow indicates a spike–HS bond rupture event. (D) Representative data on surface dwell time vs binding probability of spike VOCs and HS interactions. Solid line: single exponential function for pseudo-first-order binding kinetics. (E) Representative dynamic force spectra (mean rupture force vs loading rate) at a dwell time of 0 s. Solid line: Bell–Evans model fit. (F) Average rupture force of the spike–HS bond. Each point represents the average over each loading range for every sample tested. *P*-values were determined by one-way ANOVA tests: *p < 0.05, **p < 0.01, and ****p < 0.0001.

Noting the effect of HS on the binding kinetics on cell membrane extracts and the previous reports of interaction between HS and purified spike from VOCs,^14^ we tested the multivalent interaction of spike-decorated liposomes to surface-immobilized HS. EFA showed an overall tendency toward a faster attachment to HS of more recent VOCs. Omicron, and to a lesser extent Delta, displayed a significantly faster attachment with an increment in *k*_m_ of ∼10 times for Omicron and ∼3 times for Delta when compared to earlier VOCs (Figure 4A). The increase in the association rate is accompanied by an increase in the equilibrium surface coverage, indicating an overall higher multivalent affinity to HS for Omicron and Delta (Figure 4B). On surface-immobilized HS, we could not obtain a value for *k*_off_, as the interaction between the particles and the surface proved to be too stable, resulting in too few detachment events to confidently measure the dissociation rate constant over the duration of the experiment. A similarly large increase in the attachment rate and surface coverage was observed for Omicron UV-inactivated SARS-CoV-2 virus particles when compared to the original Wuhan strain (Figure S13), confirming the suitability of spike-decorated liposomes as virion mimics. Negligible binding was observed for the nonsulfated but negatively charged hyaluronic acid (HA), used as a negative control. This highlights the fact that the interaction between spike and HS does not solely rely on electrostatic interactions but exhibits a certain degree of specificity for the presence of specific sulfation patterns. Altogether, our results confirm the large increase in the attachment rate for Omicron virion mimics to HS compared to previous VOCs.

### AFM-Based SMFS Reveals a Complex Evolutionary Trajectory of HS Binding across VOCs

Given evidence of both HS binding to spike, its screening effect on nSLBs, and the significant variation in the HS role between VOCs, we characterized the spike–HS bond using AFM-based SMFS. In these experiments, the AFM cantilever is functionalized by immobilizing spike at a very low density on the tip apex through a flexible PEG linker. The tip is brought into contact with the HS surface to form bonds. Retraction of the cantilever at a set speed eventually causes the bond to rupture^37^ (Figure 4C). Both the distance to the surface and the force exerted by the AFM tip can be simultaneously measured, yielding force–distance (FD) curves (Figure 4C (inset) and Figure S14A). Such a curve exhibits a nonspecific adhesion peak followed by the characteristic stretching of PEG and finally the bond rupture event. The rupture events were specific to spike–HS interactions, as they were largely absent for the NTA-coated tip (i.e., in the absence of spike) against the HS surface (Figure S14B) and for spike-coated tips (Wuhan) against HA (Figure S14C). In addition, we verified that spike–HS interactions were mainly originating from single unbinding events through several observations: (i) the binding probability (BP) was <20% for spike–HS bonds^38^ (Figure S14C); (ii) the Kuhn length of the PEG linker was 0.68 ± 0.13 nm, which is in agreement with the literature value of 0.7 nm for the stretching of a single PEG chain^39^ (Figure S14D); (iii) all force histograms had a unimodal Gaussian distribution (Figure S15).

From the SMFS data, we computed the kinetic parameters of the spike–HS interaction, as summarized in Table 1. The single-molecule association rate constant () was obtained by studying the increase in binding probability with contact time (Figure 4D and Figure S16). We observed a monotonic increase in the attachment rate with the time of emergence between VOCs (Omicron > Delta > Alpha > Wuhan), in good agreement with the binding kinetics for spike-decorated liposomes to HS films. The single-molecule dissociation rate constant () was determined by plotting the unbinding forces as a function of the instantaneous loading rate (the effective linker stiffness multiplied by the retract speed, see materials and methods) and fitting them to the Bell–Evan model (Figure 4E and Figure S17). Omicron showed the lowest dissociation rate. However, in this case, a more complicated interaction was observed for the other VOCs, with higher  values, i.e., a faster dissociation, for Delta and Alpha compared to Wuhan (∼6 and ∼2 times, respectively). Taken together, the Omicron spike formed a considerably more stable bond with HS, as evidenced by a single-molecule dissociation constant () of 34 nM, 10 times lower than for Wuhan (Table 1). Surprisingly,  values for Delta and Alpha were ∼2.5 and ∼1.2 times higher than Wuhan, respectively. These results indicate significant differences in the interaction characteristics of the various VOCs with HS, with a more dynamic bond for Alpha and Delta, and a stable high-affinity interaction for Omicron. Notably, in addition to a more dynamic binding behavior, Alpha and Delta showed a significantly higher unbinding force over the whole range of the loading rate probed, compared to Omicron (Figure 4F), indicative of higher bond mechanical stability.^38−40^ All in all, SFMS analysis confirms the high affinity between Omicron and HS but also reveals a much more dynamic interaction for previous VOCs, with fast attachment and detachment kinetics.

**Table 1:** Summary of the Single-Molecule Association () and Dissociation () Rate Constants, Energy Barrier Width (*x*_β_), and Binding Affinity () of Spike VOCs for HS Measured by AFM-Based SMFS^a^

**spike VOCs**	**(10**^**5**^ **M**^**–1**^ **s**^**–1**^**)**	**(s**^**–1**^**)**	*x*_**β**_**(nm)**	**(nM)**
Omicron	4.15 ± 2.01	0.014 ± 0.007	1.08 ± 0.19	34 ± 23
Delta	3.93 ± 1.39	0.32 ± 0.22	0.75 ± 0.18	829 ± 633
Alpha	2.51 ± 0.13	0.10 ± 0.05	0.72 ± 0.09	416 ± 197
Wuhan strain	1.57 ± 0.44	0.052 ± 0.027	0.88 ± 0.03	334 ± 198

a SMFS reveals high-affinity binding to HS for Omicron and highly dynamic interaction for Alpha and Delta.

## Discussion

The emergence of new SARS-CoV-2 VOCs has been characterized by the accumulation of mutations in spike, especially within the receptor binding domain (RBD). These mutations have been shown to alter the interaction of spike with individual/isolated cellular components.^2,41^ However, little is known about their effects on the interaction with the cell surface as a whole, the interplay of its components, and how this could relate to changes in infectivity, viral tropism, and viral transmissibility. In this systematic study, we employ spike-decorated liposomes from four VOCs, to explore the kinetics of the early interaction between SARS-CoV-2 virions and the surface of susceptible pulmonary cells, and we elucidate their evolution during the COVID-19 pandemic.

We observed a notable rise in the binding of Omicron to human pulmonary Calu-3 cells, indicating the virus’ adaptation toward a more efficient attachment to the host plasma membrane of these cells (Figure 2). This increase stands in contrast to the reported reduced infectivity in Calu-3 cells in culture.^35,42^ The lack of correlation between binding and infectivity suggests an increased interaction with membrane components other than ACE2. These interactions may trap the viral particle, preventing efficient virus internalization or reducing the mobility of the virus on the cell surface, thus lowering the possibility to engage ACE2. The kinetic analysis of the attachment on Calu-3-derived nSLBs shows that the increased binding is driven by a faster attachment rate (Figure 3). Improved attachment indicates a larger availability of binding sites, which may act as decoy receptors, as has been observed for other viruses.^43^ Interestingly, we observed a similar multivalent affinity to the nSLB for Alpha and Omicron (Figure 3B–D). This stands in contrast to the increased number of Omicron particles attached to live cells. We believe that this discrepancy is the result of a higher affinity of Alpha’s RBD to ACE2 than that of the other VOCs, as reported by Han et al.^6^ Indeed, the incorporation of synthetic lipids in the nSLB dilutes the membrane material, likely improving access to ACE2 compared with the interaction with live cells. This suggests a screening effect of membrane components on ACE2 binding. These findings indicate that the increased binding observed for Omicron is due to interaction with binding factors beyond ACE2 and points toward an important and evolving role of coreceptors and ACE2 accessibility.

In our work, we further identify HS as a major membrane component engaged in modulating the interaction of SARS-CoV-2 at the cell surface. Enzymatic removal of HS from nSLBs resulted in an increase in both attachment and affinity to the surface for all VOCs but Omicron, as compared to the untreated case (Figure 3G,H). Complementing previous reports of binding between spike and HS,^10,14^ our results indicate that HS chains on the cell surface partially hide high-affinity receptors, most likely ACE2, and that the direct interaction with HS is not sufficient to compensate for the reduced accessibility of these receptors. The increased accessibility of ACE2 after the enzymatic removal of HS is also supported by the expected higher affinity measured for Alpha reminiscent of its high affinity to ACE2.^6^ Conversely, in the literature, HS removal was linked to reduction of infection due to the suggested stabilizing effect of HS binding in the “open” conformation for the RBD domain.^10^ This apparent contradiction highlights an ambivalent and possibly regulatory role of HS for early VOCs, on one side masking ACE2 and on the other allowing for a common but weak attachment point at the cell surface, which “primes” the virion prior to the interaction with the receptor.

Contrary to other VOCs, Omicron does not show any increase in association and affinity after HS removal. This results in a reduced attachment after the enzymatic treatment of nSLBs compared to that of Alpha and Wuhan, in clear contrast with the highest rate observed on untreated bilayers (Figure 3D/4B). We interpret this as the result of Omicron’s increased affinity to HS, which compensates for the reduced accessibility of ACE2. We confirmed our hypothesis by further characterizing the spike–HS interaction using HS immobilized on the surface in a glycocalyx-mimicking architecture. We report a significant (∼10-fold) increase in both the attachment rate and the number of bound particles at equilibrium compared to the original Wuhan strain (Figure 4A,B), in agreement with previous reports between isolated Omicron’s spike and HS.^14,44^ The analysis of the bond at the single-molecule level using AFM-based SMFS further revealed a high affinity (∼35 nM) for an individual Omicron’s spike–HS interaction, in the same range as the one reported for ACE2,^6,8,45−48^ confirming the role of HS as a primary attachment factor (Table 1).

The in-depth characterization of the interaction between HS and earlier VOCs revealed an overall weak binding with HS at both the multivalent and single-molecule levels. SMFS data indicate a monovalent interaction in the sub-μM range (Table 1), in agreement with previous studies, and around 2 orders of magnitude lower than what was reported for ACE2. In addition, the attachment rate of spike-decorated liposomes from Alpha and Wuhan shows only a small and nonsignificant increase compared to the negative control, indicating a marginal role in binding for these VOCs and supporting the screening role observed in nSLBs (Figure 4A,B). Furthermore, the SMFS data highlighted differences in the individual spike–glycan interaction of early VOCs with HS. Noteworthy, we report lower affinity to HS for Alpha and Delta than Wuhan, together with a more dynamic behavior, with increased association and dissociation rates. Previous studies using enzyme-linked immunosorbent assays or surface plasmon resonance and soluble spike or isolated RBD showed a small increase in the affinity of Delta to HS, similar to the results of our TIRF-based kinetic assay (Figure 4A), and no clear indication of significant variations in the attachment and detachment rates compared to the Wuhan strain.^14,44^ The reason for this discrepancy might lie in the technique used. When measuring the adsorption of proteins onto a functionalized surface, the protein establishes multiple bonds with the same or several chains, which stabilize the interaction and mask the possible rapid creation and rupture of single bonds. Using AFM-based SMFS, we directly addressed single bonds, not allowing possible rebinding and preventing avidity effects.^49^ We speculate that this dynamic interaction can promote virion diffusion through the cellular glycocalyx and facilitate virion transfer to ACE2. In addition to a more dynamic interaction, Alpha and Delta recorded the highest average unbinding force, i.e., the mechanically strongest bond, allowing the virions to better withstand external forces, such as the ones present in the respiratory tract.^50^

**Figure 5:**
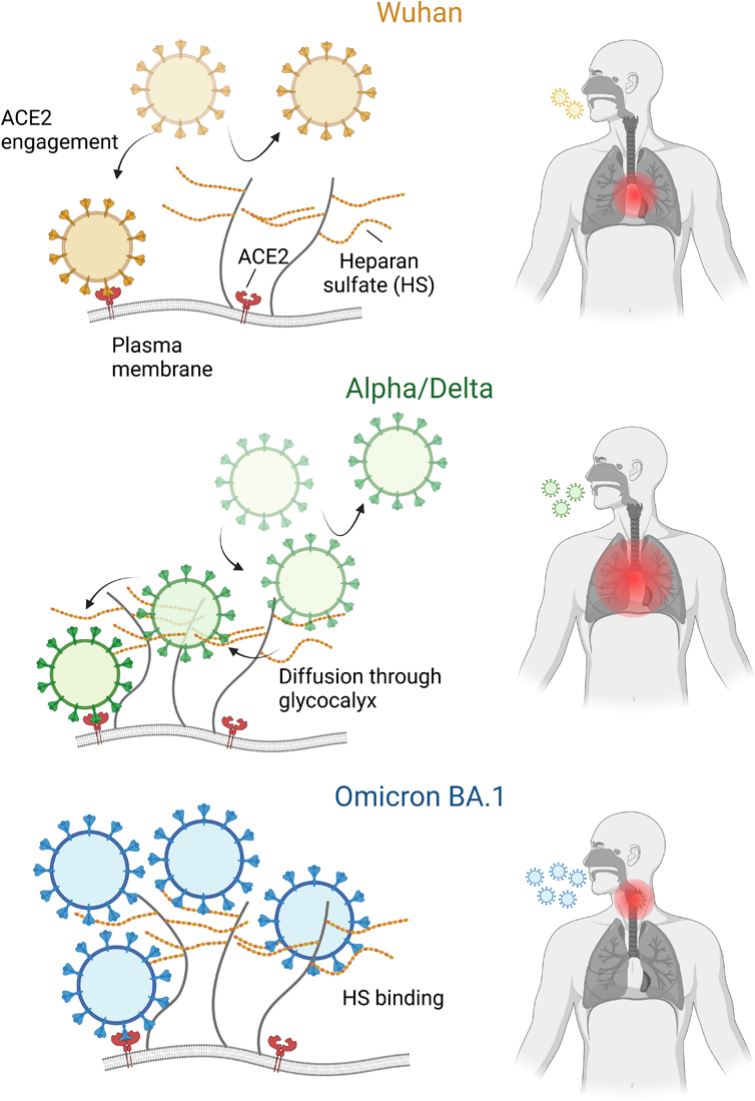
Interaction with HS could affect the VOC tropism. The high affinity of the interaction between Omicron and HS suggests efficient virus capture in the upper respiratory tract resulting in a milder disease but high shedding. For Alpha and Delta, the dynamic interaction with HS might result in more efficient diffusion through the cellular glycocalyx and ACE2 recruitment compared to Wuhan. This could contribute to higher infectivity, shedding, and disease severity.

We propose that early VOCs evolved to use HS as a first attachment point, hence the higher attachment rate, but produced only transient but mechanically stable bonds. This could allow the virion to more easily navigate through the cellular glycocalyx while sustaining the shear forces present in the respiratory tract, to eventually transfer to the ACE2 receptor, as has been proposed for other viruses.^4,50^ A sudden shift in the virus evolutionary trajectory is instead seen with Omicron. We speculate that the increased affinity to HS is one of the causes for the changes in the symptoms observed in Omicron infection, the increased transmissibility of the virus, and the reduction in infectivity at the cellular level. HS is ubiquitously expressed in tissues and highly expressed both in the nasal cavity and in the lungs.^51^ We thus speculate that the availability of binding sites in the nasal cavity and upper respiratory tract prevents the virus from traveling far in the airways and reaching the lungs in large amounts, as was the case for previous VOCs. This causes an infection of the upper respiratory tract with lower chances of lung inflammation and an overall milder disease. While the strong interaction with HS might reduce the virus mobility at the cell surface and impair ACE2 engagement and entry, the resulting accumulation in the upper respiratory tract could also represent an evolutionary advantage for the virus as the infection closer to the nose and mouth results in more efficient incorporation of viral particles in aerosols,^52^ causing highly infectious shedding and ultimately high transmissibility^53^ (Figure 5). Our study exclusively relies on pulmonary Calu-3 cells to reveal the key role of HS in regulating virus interaction dynamics at the cell surface. Because the exact chemical profile of HS at the cell surface is highly cell- and tissue-dependent,^54^ future studies using cells originating from both the upper and lower respiratory tracts promise to provide further insights into the role of HS in shaping viral tropism and infectivity.

## Conclusions

In our study, we characterize the bond formed by virion mimics carrying the SARS-CoV-2 spike protein and the plasma membrane of host cells in the early stages of the virus entry with the aid of well-defined biomimetic models and biochemical and biophysical techniques. We describe how this interaction evolved with the emergence of new VOCs and elucidate the role of HS. We begin from observations in a highly complex system: the binding of virion mimics on Calu-3 cells. We then progressively reduce the complexity of the system to allow an in-depth elucidation of the characteristics and kinetics of the bond formed, at both the single-particle and single-molecule level, elucidating the role of HS in several VOCs.

By addressing both the molecular properties of the interaction with spike and the role that they play in virus attachment, we provide insights into how viral attachment and the interaction with HS could be linked to a shift in the tropism of VOCs, both in transmissibility and in the resulting symptoms of the infection. We highlight the importance of accounting for the complexity of the plasma membrane when investigating the role of membrane components in viral attachment as well as the synergy necessary between cellular and molecular models and techniques. This illustrates the importance of multiscale and transversal studies to elucidate the biological role of physical and chemical interaction. Given the flexibility of our platform and approach, we envision our study to be expanded in the future to address other proposed coreceptors and new emerging VOCs, building toward a complete picture of the concurring molecular interactions exploited by SARS-CoV-2 during host engagement and entry.

## Supporting Information Available

Additional details for the materials and methods section, supporting figures referenced in the main text regarding spike-decorated liposomes and nSLB characterization and additional kinetic data from EFA and AFM-based SMFS, and summary of non-normalized data from EFA (PDF)
