# Supplementary material for: Variant-Specific Interactions at the Plasma Membrane: Heparan Sulfate’s Impact on SARS-CoV-2 Binding Kinetics
Source: Anal Chem. 2025 Feb 20;97(8):4318–28. doi: 10.1021/acs.analchem.4c04283 (PMC11883730; doi:10.1021/acs.analchem.4c04283)
Supplement: Supplementary file 1 — ac4c04283_si_001.pdf [file ac4c04283_si_001.pdf]

Supporting information

Variant-specific Interactions at the Plasma Membrane: Heparan Sulfate's Impact on SARS-CoV-2 Binding Kinetics

Dario Valter Conca[1,2,3*], Fouzia Bano[1,2,3], Małgorzata Graul[1,2,3], Julius von Wirén[1,2,3], Lauriane Scherrer[1,2,3], Hudson Pace[1,2,3], Himanshu Sharma[2,3,4,5], Justas Svirelis[6], Konrad Thorsteinsson[1,2,3], Andreas Dahlin[6], Marta Bally[1,2,3*]

[1] Department of Clinical Microbiology, Umeå University, Sweden

[2] Wallenberg Centre for Molecular Medicine (WCMM), Umeå University, Sweden

[3] Umeå Centre for Microbial Research (UCMR), Umeå University, Sweden

[4] Department of Medical Biochemistry and Biophysics, Umeå University, Sweden

[5] Laboratory for Molecular Infection Medicine Sweden (MIMS), Umeå University, Umeå, Sweden

[6] Department of Chemistry and Chemical Engineering, Chalmers University of Technology, Sweden

* Correspondence: Dario Valter Conca, dario.conca@umu.se; Marta Bally, marta.bally@umu.se

Content

Supporting material and methods 2

Supporting figures 12

Supporting tables 23

Supporting references 25

Supporting material and methods

Small unilamellar vesicles (SUVs) production

Lyophilized lipids were dissolved in chloroform at concentrations between 2 and 50 mg/ml, mixed to the desired molar concentration and dried first under gentle nitrogen flow and then under vacuum overnight. The liposomes containing NTA-lipids were then resuspended in 1 ml phosphate buffer saline (PBS, Medicago AB, Sweden), or HEPES buffer saline (HBS, 150 mM NaCl, 10 mM HEPES) at a concentration between 0.5 and 2 mg/ml. SUVs were formed by extruding at least 21 times through a polycarbonate membrane, using a mini extruder (Avanti Polar Lipids; 610020), as previously described[1]. Pure POPC vesicles, POPC:bioDOPE (95:5 molar ratio), POPC:PEG (99:1 molar ratio) and POPC:OG-DHPE (99:1 molar ratio) used for the creation of supported lipid bilayers (SLB) were extruded through a 50 nm membrane. For the production of spike-decorated liposomes, the POPC:DGS-NTA:Liss-Rhod mixture (99-X:X:1 molar ratio, where X is the percentage of DGS-NTA lipids) was flash frozen and thawed 5 times by alternate immersion in liquid nitrogen and 37ºC water bath and then extruded at least 21 times through a 100 nm membrane. All stocks were stored at 4ºC for up to three months.

Characterization of the size distribution of spike-decorated liposomes via DLS

5 µl of spike-decorated liposomes were collected after incubation with Capto Core 700 beads (see Experimental section in the main text) and diluted to a final volume of 50 µl in PBS. The solution was added to a 40 µl cuvette (ZEN0040, Malvern Panalytical Ltd, UK) and measured using dynamic light scattering with a Zetasizer Nano S (Malvern Panalytical Ltd). The data were analyzed using Zetasizer proprietary software (Malvern Panalytical Ltd). Two independent measurements were performed for each sample.

Quantification liposome size and concentration via nanoparticle tracking analysis

Nanoparticle tracking analysis was carried out on POPC:DGS-NTA:Liss-Rhod (98:1:1) liposomes diluted to three concentrations 1, 0.5, and 0.25 µg/mL in HBS and analyzed using a NanoSight NS300 instrument (Malvern Panalytical Ltd, UK) equipped with a 488 nm laser. Each sample was analyzed under continuous flow (flow rate = 30 (no units)) and five 60 s movies were collected and averaged. The data were analyzed using NanoSight proprietary software (Malvern Panalytical Ltd). The particle concentration was measured to be $3.5 \pm 0.1 \cdot 10^{12}$ particles/ml, for a nominal lipid concentration of 2 mg/ml, and the average particle size was 126 ± 3 nm.

Western blot quantification of spike capture by liposomes

We used Western blotting for the quantification of the amount of spike protein captured by the NTA liposomes. 20 µl of spike-decorated liposomes was mixed with 4 µl of SDS and 1 µl of 1 M imidazole

to dissolve the vesicles and detach the spike. 5 µl of 6x Laemmli buffer was added and the samples were incubated at 95ºC for 10 min. The samples were then carefully loaded into a precast 15-well bis-tris SDS gel (NW04125BOX, Thermo Fisher Scientific). In addition, a serial dilution of soluble spike from 1 to 60 ng was prepared and loaded on the same gel as the liposome lysates to allow for quantification of the spike content of the liposome solution (Fig. S1). Gel electrophoresis was performed at 200 V for 35 min in 1X NuPAGE™ MES SDS Running Buffer (NP0002, Thermo Fisher Scientific). A PVDF (polyvinylidene difluoride) membrane was prewetted in methanol, and proteins were transferred in transfer buffer solution (10% 10x Tris/Glycine Buffer (1610734, Biorad), 20% Methanol and 70% milli-Q H_2O) using a constant current of 300 mA for 45 min in a Mini Trans-Blot® Cell (Biorad). The membrane was blocked in 5% fat-free milk in PBS + 0.05% Tween-20 (PBST) for 1 hour and then incubated with anti-His-tag (34660, QIAGEN, Hilden, Germany) diluted 1:1000 in 5% milk in PBST at 4ºC overnight. After 3 washes in PBST, the membrane was incubated in a 1:2000 dilution of horseradish peroxidase (HRP) conjugated anti-mouse antibody (AB_228307, Invitrogen) in 5% milk in PBST. Finally, the membrane was thoroughly washed and the chemiluminescence signal was imaged using SuperSignal™ West Pico PLUS chemiluminescence substrate (Thermo Fisher Scientific) in an Amersham™ Imager 680 blot reader (Cytiva, Chicago, USA).

The chemiluminescence images were analyzed using ImageJ. A calibration curve relating the band intensity with known spike content was created from the soluble spike dilution and used to quantify the spike content in the liposome lysate for each experiment. The limit of detection of this method was determined by serial dilution of soluble spike protein to be ~1 ng, with a linear range between 5 and 60 ng. Spike quantification was performed in 4 independent experiments, including all variants. All measured values fell in the linear range of the assay (maximum: 60.6 ng, minimum: 30.0 ng). The average spike mass in the sample was 40.9 ± 10.0 ng (mean ± standard deviation, N=14), which corresponds to a spike concentration in the liposome solution (c_{spike}) of 2.0 µg/ml.

Immunostaining of spike-decorated liposomes

Immunostaining of spike-decorated liposomes was performed using the following protocol. Spike-decorated liposomes containing rhodamine lipids and carrying the Wuhan variant were diluted 1:10 and adsorbed on a clean glass surface for 1 minute. After washing in PBS, the glass was blocked with a 2% solution of BSA in PBS (blocking solution) for 1 h. Anti-SARS-CoV-2 spike antibody (MA5-35946, Thermo Fisher Scientific) was then added to a final concentration of 1:100 in blocking solution and incubated for 1 h. After thorough rinsing, the adsorbed particles were stained with an anti-mouse Alexa-488-conjugated secondary antibody at a final dilution of 1:250 for 30 minutes. After rinsing, the slides were imaged using the Nikon Eclipse Ti2 microscope in total internal reflection fluorescence microscopy (TIRFM) configuration using the 488 nm laser at 40% power and 500 ms exposure time and the 561 nm laser at 20% power and 300 ms exposure. 12 images for each condition were acquired

and analyzed. Particles without the addition of spike and particles functionalized in the absence of $NiCl_2$, i.e. impairing the interaction between NTA and His-tag, were used as negative controls.

The colocalization between the fluorescent signal from the rhodamine conjugated lipids incorporated in the liposomes and the secondary antibody was analyzed using an in-house MATLAB script, using an intensity threshold for particle detection of 1000 in both channels. Signals are considered colocalized if the distance between centroid in the 488 and 561 nm channels is smaller than 2 pixels (320 nm), to account for possible sample movement between acquisitions, localization uncertainty due to image noise and alignment of the two channels.

Liposome concentration measurement with bouncing particle analysis and determination of the average number of spike per liposome

The particle concentration in solution was measured for every sample using "bouncing particle analysis" (BPA) as described previously[2]. In brief, the sample was first bleached to remove the signal from particles on the surface, and then a 30-60 s video was acquired at 10 fps in TIRFM configuration. The videos were analyzed in the same way as for the kinetic analysis, but, in this case, only particles entering the TIRFM volume but not binding to the surface (residence time lower than 1 s) were considered. Since the particles freely diffuse in the buffer, the number of the recorded particles is proportional to their concentration in solution, or the absolute concentration determined via a calibration curve (Fig. S2). In this way, the relative concentration of the particles between samples can be measured. The linearity between the BPA results and particle concentration was demonstrated by measuring a serial dilution of fluorescently labelled SUVs on an inert POPC bilayer, Fig. S2. The SUV stock was diluted in PBS from 500 to 8000 times in two independent experiments. Four areas were imaged per experiment and condition.

The number of particles observed in each frame was related to the absolute number of particles in the liposome stock as calculated by nanoparticle tracking analysis as:

$$C_{\mathrm{BPA}} = \frac{\text{particle concetration in stock}}{\text{dilution factor} \cdot \text{detected particles per frame}},$$

where C_{BPA} is the conversion factor. The value of C_{BPA} was calculated as the slope of the linear fit of the results of the serial dilution to be $8.9 \cdot 10^7$ ml^{-1}, as shown in Fig. S2.

The concentration of spike-decorated liposomes in solution in each experiment (c_{sol}) can be then determined as:

$$c_{sol} = C_{\mathrm{BPA}} \cdot \text{ detected particles per frame.}$$

By measuring both the liposome concentration in solution and the total spike concentration of a sample, c_{spike} (as described in "Western blot quantification of spike capture by liposomes"), it is possible to determine the average number of spike protein per liposome as:

$$n_{\text{spike}} = \frac{c_{\text{spike}}}{c_{\text{sol}}} \frac{\text{MW}}{\text{NA}},$$

where MW is the molecular weight of a spike trimer (~540 kDa)[3], and NA is Avogadro's number. We determined n_{spike} to be 69 ± 19 (mean ± standard error of the mean) from 3 independent experiments.

Functionalization AFM tips for AFM-based SFMS experiments

AFM probes (MSCT, Bruker) were treated in UV/ozone cleaner for 30 mins. The cleaned cantilevers were then exposed to APTES (3-Aminoproyl)triethoxysilane) and triethylamine using the gas-phase salinization reaction[4]. Next, the cantilevers were incubated in 0.5 ml of chloroform containing 1 mg of NHS-PEG-Acetal MW 2k (Creative PEG, USA) and 30 µL of triethylamine for 2 h. Following washing with chloroform (5 min, 3x), the cantilevers were dried with nitrogen and either stored under nitrogen until use or functionalized instantly with Tris-NTA-amine linker (BR1001101, biotechrabbit, Germany). For the functionalization with Tris-NTA-amine linker, the cantilever was immersed in 1% w/v citric acid in milli-Q water for 10 mins. After washing with milli-Q water (5 min, 3x), the cantilever was incubated in the solution containing 0.71 µg/ml of Tris-NTA-amine in PBS and 2 µL of freshly prepared sodium cyanoborohydride ($NaCNBH_3$) solution (13 mg of $NaCNBH_3$ dissolved in 20 µL of 20 mM NaOH and 180 µL of milli-Q water) for 30 mins. The coupling reaction was then quenched with 5 µL of 1 M (pH 8.0) ethanolamine solution and incubated for 10 min. The cantilever was then carefully rinsed 4 times with 1 ml PBS, dried with nitrogen, and instantly mounted on the AFM cantilever holder. Next, 100 µL of 10 mM $NiCl_2$ in HEPES buffer was carefully added to the cantilever and incubated for 15 mins. After rinsing twice with 1 ml HEPES and twice with 1 ml PBS, the cantilever was incubated with His-tagged spike at the final concentration of 1.5 µg/ml for 10 mins to ensure the coupling of a very low concentration of spike proteins on the tip apex. All chemicals used for AFM tip functionalization were purchased from Sigma-Aldrich unless otherwise stated.

Multi-Parametric Surface Plasmon Resonance (SPR)

To obtain silica-coated chips for SPR, SPR chips with ~2 nm chromium (Cr) and 50 nm gold (Au) were prepared by electron-beam-heated physical vapor deposition (Lesker PVD 225) on glass substrates (20 x 12 mm; BioNavis), cleaned beforehand with RCA-2 (1:1:5 vol. conc. of HCl:H_2O_2(30%):milli-Q H_2O) at 80°C and 50 W O_2 plasma at 250 mTorr. An additional thin silica layer (~11 nm) was deposited on top of the gold layer with atomic layer deposition (Oxford FlexAL) at 300°C by using a bis(t-butylamino)silane (BTBAS) as a precursor and oxygen as processing gas. The sensor surfaces were cleaned in a UV/ozone chamber for at least 30 min before use.

The SPR Navi^{TM} 220A instrument (BioNavis) is equipped with three lasers (wavelengths of 670 nm, 785 nm, and 980 nm), which induce different decay lengths of sensing fields. The SPR spectra can be acquired in parallel from 2 flow channels in a polyether ether ketone flow cell, exposed to the metal side of the sensor. We used PBS as running buffer and ran at ∼5 – 20 µL/min during the experiments. Once a stable baseline was achieved, injections of necessary solutions were automated by using an autosampler and a 96-well plate. The temperature was kept at 25°C in all the experiments. The quantification of thickness, mass coverage or surface grafting density of layers, adhered on silica-coated gold sensors were estimated as reported before (see next section)[5–7]. In brief, the SPR spectra after silica coating were fitted with Fresnel models to retrieve the dry film thickness by using a custom MATLAB (MathWorks, Natick, MA, USA) code[8]. In liquid, the surface coverages (Γ) were estimated from independently determined parameters (e.g. molar refractivity dn/dC, plasmonic field decay length δ, etc.) as described previously[9], see next section.

The conditions for binding poly-His-tag spike onto NTA-bilayers in SPR experiments were the following: (i) the SLB was formed by injecting of SUVs containing 0.25% or 1% of NTA functionalized lipids at concentration of 100 µg/ml and a flow rate of 20 µL/min, (ii) spike was injected at a concentration of 18.33 µg/ml and a flow rate of 5 µl/min for 1 h.

Quantification of spike optical mass by SPR

For quantifying the number of adsorbed biomolecules and thus surface grafting density from the recorded SPR sensogram signal ($\Delta\theta$) presented in Fig. S8D we have used the following equation:

$$\Gamma = {}^{\Delta\theta\delta}/_{S_0 b}$$

Here b is the increase in refractive index per mass concentration of the binding species (0.18 ml/g for proteins and 0.17 ml/g for supported lipid bilayers). S_0 is the bulk sensitivity of the instrument (angular shift per refractive index increment), which was estimated from Fresnel models at 670 nm to be 131 degrees[5]. δ is the decay length of the evanescent field, which is calculated from the dispersion relation and the field distribution of the surface plasmons[10]. Considering experimentally determined parameters for the Cr and Au thickness and permittivity, we have δ = 228 nm for our system.

Using this model, from the SPR sensograms in Fig. S8D we calculated the spike density on a bilayer with 0.25% molar concentration of NTA to be 62 ng/cm^2, which corresponds to a trimer area density of 920 trimers/µm^2. Assuming a spherical liposome of diameter 125 nm, this corresponds to ~45 trimer per particle, comparable to what was reported for SARS-CoV-2 virions[11,12].

Quartz crystal microbalance with dissipation monitoring measurement

Quartz crystal microbalance with dissipation monitoring (QCM-D) measurements were performed with an AWS X4 QCMD system (AWSensors, Valencia, Spain) using silica-coated sensors (AWS SNS

000049 A, AWSensors). The sensors were cleaned in SDS (2% w/v) for 30 minutes and then rinsed twice in 99.9% ethanol and 10 times in milli-Q water and then dried under nitrogen flow. The sample chamber and tubing were washed for 30 minutes in Cobas cleaner (20754765322, Roche, Basel, Switzerland), rinsed with abundant milli-Q water and dried under nitrogen flow. The sensor was then mounted into the chamber and kept under HBS flow. The SLB was formed using POPC:DGS-NTA:LissRhod (99-X:X:1 molar ratio, where X is the percentage of DGS-NTA lipids) vesicles at a final concentration of 100 µg/ml in 20 mM $NiCl_2$ in HBS. The vesicles spontaneously ruptured on the silica surface, forming an SLB. After rinsing the sample with HBS, 50 µl of spike solution (20-100 µg/ml) in HBS was injected; the protein solution was incubated for >30 min in static conditions. The chamber was then rinsed in PBS and the spike detached using a 0.5 M solution of imidazole in milli-Q water, to verify specific attachment via NTA-His-tags. All solutions were injected at a flow rate of 20 µl/min. In between measurements, the sensors were cleaned in SDS and water, stored in SDS or dried over nitrogen flow, and reused up to 10 times.

To test the specificity of the interaction between the poly-His-tag on the spike and the NTA bilayer, spike solution (20 µg/ml) was incubated with anti-His-tag antibody (34660, QIAGEN) at 100 µg/ml for 30 minutes before injection over the NTA bilayer.

Cryogenic electron microscopy (Cryo-EM) of spike-decorated liposomes

Spike-decorated liposomes were produced using 4 times the concentration of liposomes and protein reported in the Experimental Section of the main text, and without the final filtration through MicroSpin™ S-400 HR columns to maximize the sample concentration. On the same day, the liposomes were applied to glow-discharged Qfoil R2/2 CU 300 or Lacey carbon 200 mesh grids (with a 2 nm carbon layer) and blotting was done using a Leica GP2 system set to the following settings: delay time of 1 minute, temperature at 22 °C, humidity at 95%, 10 s blot time, followed by plunge freezing in liquid ethane. Cryo-frozen grids were imaged on 200 keV Thermo Scientific Glacios Microscope equipped with a Falcon 4i Direct Electron Detector. The micrographs were acquired with the software EPU (Thermo Scientific) in counting mode at magnified pixel sizes of 0.95 Å, at doses of 40–50 e-/sqÅ, at defocus values ranging from −3 to −5.0 µm.

Images of single particles were denoised using a band-pass spatial filter in ImageJ (band: 30 to 500 pixels or 2.8 to 47.6 nm) to improve visibility of the spike trimers.

Production of native membrane vesicles (NMVs) from Calu-3 cells

Calu-3 cells were grown in DMEM + 10% FBS + 1% PenStrep and expanded 1 in 4 roughly every week. 20 T-175 flasks were harvested to produce the stock used in this study, following a protocol adapted from previous studies[14]. In brief, cells were washed 3 times in ice-cold PBS and then

mechanically detached using a cell scraper in harvest buffer (PBS + protease inhibitor, cOmplete™, EDTA-free Protease Inhibitor Cocktail, 04693132001). The harvested cells were pelleted via centrifugation at 600×g for 10 min and then disrupted with a CF1 continuous cell disruptor (Constant Systems, UK). Nuclei and large organelles were pelleted at 2,000×g for 10 min, and the supernatant was collected and centrifuged at 6,000×g for 20 min to remove mitochondria. Finally, the native membrane vesicles (NMVs) were collected by centrifugation at 150,000×g for 90 min. Vesicle-containing plasma membrane material was purified using a sucrose gradient. The pellet was resuspended in harvest buffer and mixed with an equal volume of 80% weight/volume (w/v) sucrose solution. It was then layered with 30% w/v and 5% w/v sucrose solutions in harvest buffer and the gradient was centrifuged at 273,000×g for 2.5 h. NMVs raise to form a clear band between 5% and 30%, which was harvested, aliquoted, flash-frozen, and stored at -80ºC. In all steps, the cellular material was maintained on ice or at 4ºC. NMV material was quantified using a FRET assay developed by us and described previously[15].

Characterization of Calu-3-derived NMVs via Western blot

The NMV material was compared to full cell lysate using Western blot. Confluent Calu-3 cells growing on a well of a 6-well plate were washed with PBS and incubated with 300 µl of lysis buffer (1% Triton-X100, 50 mM Tris HCl, 150mM NaCl, pH=8.0) for 30 min in RT. The cell lysate was collected, centrifuged 20 min, 13000 rpm, 4ºC to remove cell debris and stored in -70ºC. The protein concentration in the prepared lysate and in NMV material was measured by absorbance at 280 nm using a DS-11 spectrophotometer (DeNovix, Wilmington, DE, USA) to guarantee equal total protein loading in the gel. 60 µg of total protein was loaded for each sample.

The Western blot was performed as described above in "Western blot quantification of spike capture by liposomes". To characterize the NMV composition anti-hACE2 (AF933, R&D Systems), anti-tubulin (DM1A clone, 62204, Invitrogen), anti-E-cadherin (610181, BD Biosciences, NJ, USA), and anti-GAPDH (G9545, Sigma) antibodies were used at 1:1000 dilution. For detection, 1:2000 dilution of horseradish peroxidase (HRP) conjugated anti-mouse (AB_228307, Invitrogen), anti-rabbit (A16104, Invitrogen) or anti-goat (ab6885, Abcam) antibodies were used.

Formation of native supported lipid bilayer (nSLB) from Calu-3 cells and heparinase treatment

nSLBs from Calu-3 cells were formed as described previously[14]. In brief, NMVs from Calu-3 cells were mixed with PEG-POPC vesicles to a 12.5:87.5, diluted in PBS to a final concentration of 200 µg/ml and sonicated for 30 min at 40ºC in a bath sonicator (37 kHz, Elmasonic S40H, Germany). Tracer vesicles, 99:1 POPC:OG-DHPE, were then mixed to a 1:1000 weight ratio before nSLB formation. No.1 microscope borosilicate glass cover slides (diameter: 22 mm, VWR, 631-0158P) were cleaned by boiling for 2 h in a 10% v/v solution of 7x detergent (MP Biomedicals, CA) in milli-Q water, rinsed in abundant milli-Q water and stored in milli-Q water for up to 3 days. Before use, the slides were rinsed

in milli-Q water, dried under nitrogen and treated in a UV/ozone oven (ProCleaner™ Plus, BioForce Nanosciences, Virginia Beach, VA, USA) for at least 30 min. Home-made PDMS wells with a sample volume of ~10 µl, cleaned in SDS, rinsed in milli-Q water and dried under nitrogen flow, were attached to the clean glass surface and 5 µl of PBS was added to each well. The vesicle mixture was added to the wells to a final concentration of 100 µg/ml. Bilayer formation was observed using TIRFM or inspected for unruptured tracer vesicles after rinsing with PBS.

The NMVs:PEG-POPC ratio to be used in the experiments was established by selecting the highest concentration of NMVs that ensure the formation of a continuous and mobile lipid bilayer with few to no tracer vesicles left after formation.

Enzymatic removal of HS from nSLB was performed by incubating the already formed nSLBs with a cocktail of Heparinase I and III (2 units/ml each) in digestion buffer (20 mM Tris-HCl, 4 mM $CaCl_2$, 100 mM NaCl, 0.01% w/v BSA) for 1 hour at room temperature. The nSLBs were then rinsed in PBS before the addition of spike-decorated liposomes or antibody staining.

Fluorescence Recovery After Photobleaching (FRAP) of nSLBs

nSLBs were formed in PDMS wells on clean coverslips as described above in section "Formation of native supported lipid bilayer from Calu-3 cells and heparinase treatment", except that the nSLBs were made from 12.5% hybrid vesicles containing Rho-PEG-POPC vesicles (1 mol% Rhodamine-DOPE, 0.5 mol% PEG5Kceramide, 98.5%POPC) rather than non-fluorescent PEG-POPC vesicles and no tracer vesicles were added. The resulting fluorescent nSLBs were imaged with the Nikon Eclipse Ti2 microscope using the 561 nm led line from the Spectra III illumination source. The nSLBs were bleached using the 561 nm laser line for 100 ms and the recovery of the nSLBs fluorescence was monitored with images taken every 1 s for 5 min. The diffusion coefficient and percentage recovery were calculated using a method and MATLAB scripts described previously by Jönsson et al[16]. The diffusion coefficient and percentage recovery were 2.33±0.09 $\mu m^2/s$ and 97±1%, respectively. The results are the average of 6 measurements performed in two independent experiments.

Immunostaining nSLBs on coverslips

nSLBs were formed in PDMS wells on clean coverslips as described above in "Formation of native supported lipid bilayer from Calu-3 cells and heparinase treatment". Heparinase I and III treatment was done as described above. After washing in PBS, the nSLBs were stained using anti-HS (370255-S, AMSBIO, United Kingdom) at a concentration of 1:100 in 0.2% w/v BSA in PBS at room temperature for 1 h. After washing, the wells were incubated with a 1:200 dilution of Alexa488-conjugated rabbit anti-mouse IgG/M (A-10680, Thermo Fisher Scientific) in 0.2% w/v BSA in PBS for 1 h. Finally, the wells were washed 3 times in PBS and the fluorescence signal was measured with the Nikon Eclipse Ti2 microscope. For background subtraction, a non-treated nSLB was exposed to secondary antibody

only. Operating in TIRF mode with a 488-laser illumination, 20 images were taken of each nSLB and the top 10 images, in terms of average pixel intensity, were then averaged together to provide the intensity value reported across at least two independent wells per condition (Fig. S5).

Production of nSLBs on glass beads and flow cytometry

To produce nSLBs on silica beads, we adapted the protocol from the production of planar nSLBs. Acid-cleaned silica beads (microParticles GmbH, Berlin, Germany) were washed 3 times in PBS and then added to the vesicle solution, 5 µl of beads stock solution in 50 µl of hybrid vesicles, followed by incubation for 1 hour at room temperature under agitation. The beads were then washed 3 times in PBS and collected via centrifugation at 1,000×g for 1 minute. Enzymatic removal of HS was performed as described above, incubating the beads for 1 hour at 37°C. After washing in PBS, the beads were stained using anti-HS (370255-S, AMSBIO, United Kingdom) or anti-ΔHS antibody (370260-S, AMSBIO), which recognize heparan sulfate chains or the epitope resulting from heparinase cleavage respectively, at a concentration of 1:100 in 0.2% w/v BSA in PBS at room temperature for 2 h. After washing, the beads were incubated with a 1:200 dilution of Alexa488-conjugated rabbit anti-mouse IgG/M (A-10680, Thermo Fisher Scientific) and Alexa488-conjugated anti-mouse IgG1 (A21202, Thermo Fisher Scientific) for the HS and ΔHS staining, respectively, in 0.2% w/v BSA in PBS for 2 h.. Finally, the beads were washed 3 times in PBS and the fluorescence signal was measured with a ZE5 cell Analyser (Bio-Rad). The flow cytometry data were analyzed using FlowJo (BD, Franklin Lakes, NJ, USA).

Immobilization of glycosaminoglycans on supported lipid bilayers

To immobilize HS for TIRFM experiments, borosilicate glass coverslips were prepared as for nSLB formation. SLBs were formed by spontaneous rupture of SUVs onto the clean glass surface. POPC:bioDOPE SUVs were prepared at a molar ration of 95:5 and diluted to a concentration of 100 µg/ml in PBS; 5 µl were added to each well and incubated for 30 min. The wells were rinsed, and streptavidin solution was added at a final concentration of 40 µg/ml. After 15 min of incubation, the wells were washed and incubated with 2% PFA for 10 minutes to crosslink streptavidin and prevent its lateral diffusion across the bilayer. After rinsing, end-on biotinylated GAGs were added at a final concentration of 40 µg/ml and incubated for 30 min to achieve full coverage of the surface. Finally, the wells were rinsed before the addition of spike-decorated liposomes.

All steps were performed at room temperature and all washing steps consisted of removing 5 µl of solution and washing 7 times with 10 µl of PBS, always leaving 5 µl of solution in the well to prevent it from drying.

Production of fluorescent UV-inactivated SARS-CoV-2 virus particles

SARS-CoV-2 virus particles were produced by infecting Vero E6 cells at an MOI of 0.1. The supernatant was collected 48-72 hours after infection, and virus was inactivated by irradiation with

UVC light with a total energy of 500 mJ using a UVP crosslinker CL-3000 (Analytic Jena, Jena, Germany). Inactivation was confirmed by plaque assay (data not shown). The virus particles stained with SP-DilC18(3) dye (D7778, Thermo Fisher Scientific), purified and concentrated by ultracentrifugation through a sucrose gradient collecting the interface between 40% and 60% sucrose solutions (w/v) and stored at -80°C until use.

Safety Statement

No unexpected or unusually high safety hazards were encountered.

Supporting figures

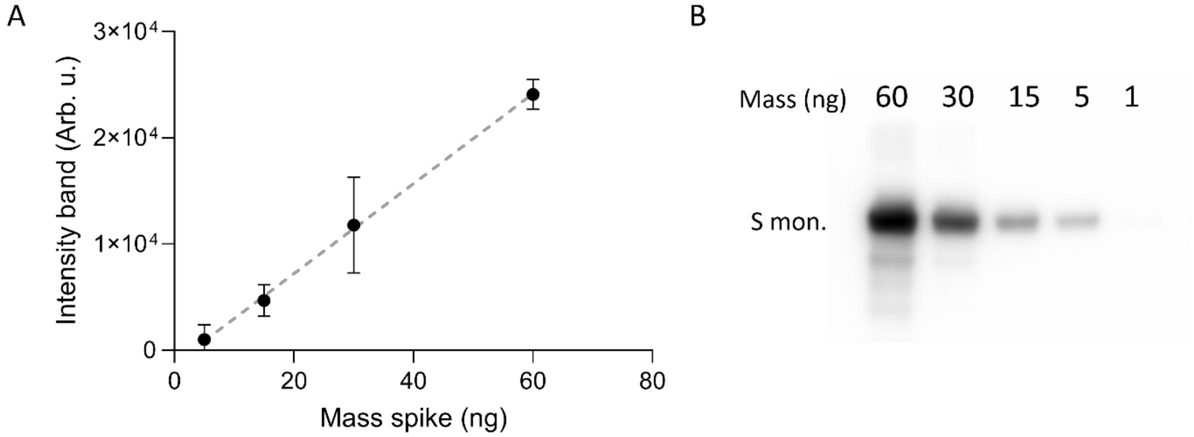

Figure S1 A. Calibration curve used to quantify the spike content of lysate of spike-decorated liposomes via Western blot, showing the linear relationship between the intensity of the chemiluminescence signal and the spike content. The black dots show the average of 4 independent experiments, the error bars indicate the standard deviation of the measurements, and the dashed grey line is the linear fit (y = 423.2 x – 1247, R squared: 0.939). **B.** Representative chemiluminescence image of one of the calibration curve used in A (S mon.: spike monomer).

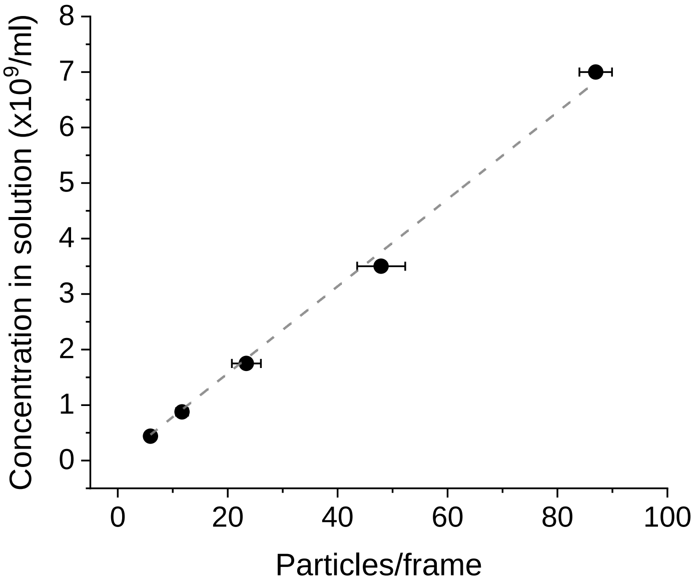

Figure S2 Number of newly detected particles per second in a serial dilution of fluorescently labelled liposomes on a POPC bilayer. The black dots show the average of 5 measurements collected in 2 independent experiments, the error bars indicated the standard deviation of the measurements and the dashed grey line the linear fit passing through the origin ($y = 8.85 \cdot 10^7 x$, R squared: 0.998).

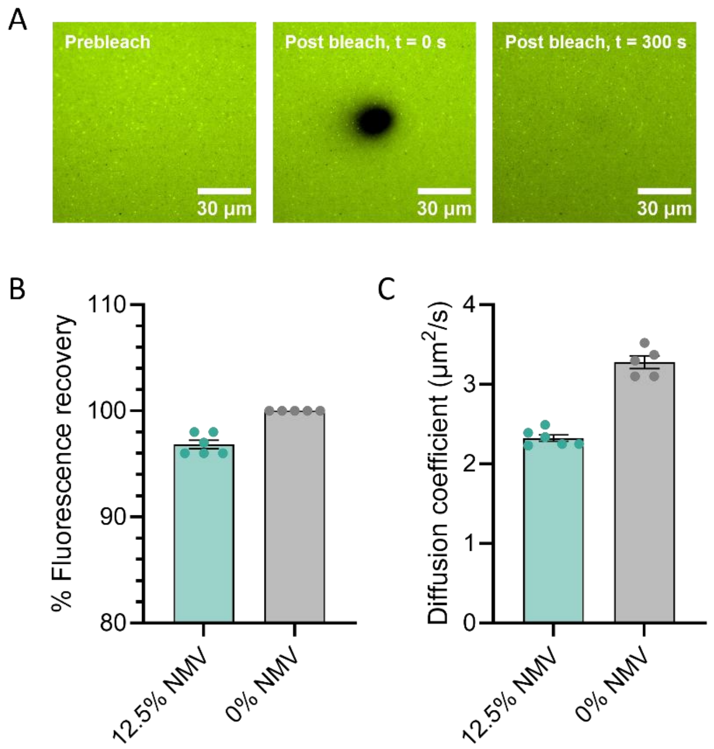

Figure S3 A. Images of a nSLB (12.5% Calu-3 material) formed from SUVs containing 1% rhodamine-conjugated lipids before (left), immediately after (center) and 300 s after photobleaching (right). **B.** Quantification of recovery after 300 s showing nearly complete recovery for nSLB (97±1%), compared to the complete recovery observed for a pure POPC bilayer (0% NMV). **C.** Diffusion coefficient of the rhodamine-conjugated lipids in the nSLB (12.5% NMV, 2.33 ± 0.09 $\mu m^2/s$) and in a pure POPC bilayer (3.27±0.04 $\mu m^2/s$). Each dot represents a single FRAP experiment, collected from 2 independent experiments. Error and error bars indicated standard error of the mean.

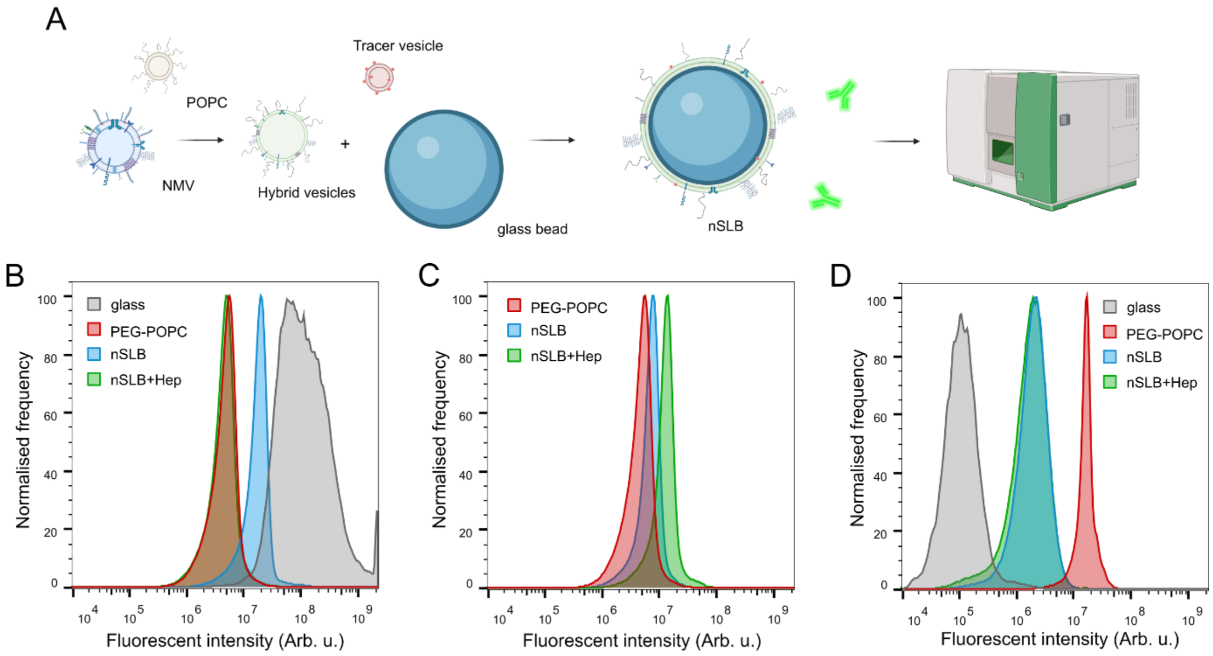

Figure S4 A. Schematics showing the process of forming nSLB on glass beads, antibody staining and final readout using flow cytometry. **B.** Flow cytometry signal from antibody staining of nSLB-coated beads for heparan sulfate, showing the signal reduction to the level of the negative control (PEG-POPC) after heparinase treatment (nSLB+Hep). Naked glass beads show a high signal due to non-specific adsorption of the secondary antibody. **C.** Flow cytometry signal from antibody staining of nSLB-coated beads against the cleavage site of heparinase-treated heparan sulfate. **D.** Flow cytometry signal from the fluorescent lipids incorporated into the nSLB, showing the presence of the nSLB on the beads. Fluorescent lipids are incorporated by adding vesicles containing 1% OG-DHPE to the PEG-POPC:NMV solution in a 1:500 v/v ratio. Pure PEG-POPC bilayers without the addition of membrane material appear brighter due to more efficient incorporation of tracer vesicles during the bilayer formation.

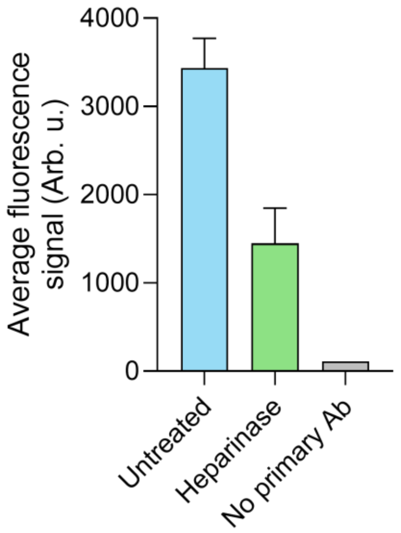

Figure S5 Anti-HS antibody staining of nSLB before and after heparinase treatment. The error bars show the standard error of the mean. The data are the result of three repeats for heparinase treatment, two for the untreated control obtained in two independent repeats. A single repeat was performed for the no primary antibody control.

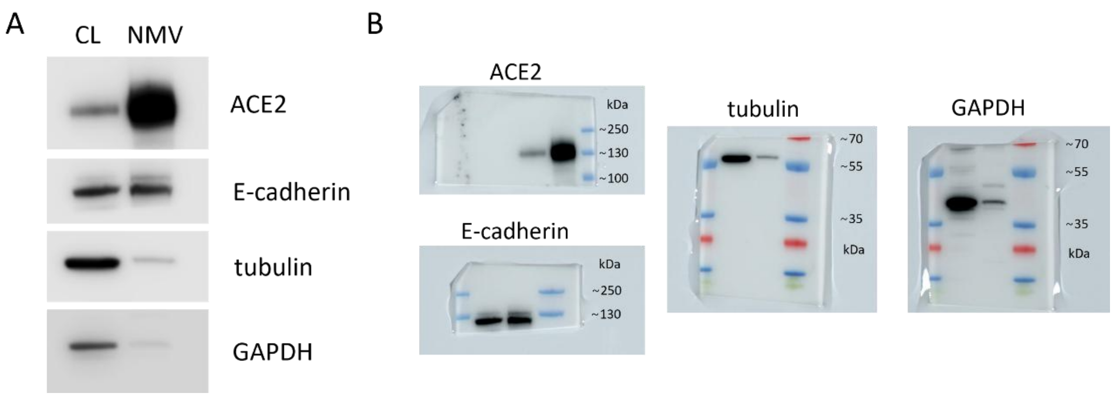

Figure S6 A. Western blot showing the presence of plasma membrane proteins (E-cadherin), enrichment of ACE2 and clear reduction of cytoplasmic protein like tubulin and GAPDH in native membrane vesicles from Calu-3 cells (NMV) when compared to cell lysate (CL) of the same cell line. 60 µg of total protein was loaded in each sample. **B.** Original Western blots used in A including protein ladder (PageRuler Plus, Thermo Scientific).

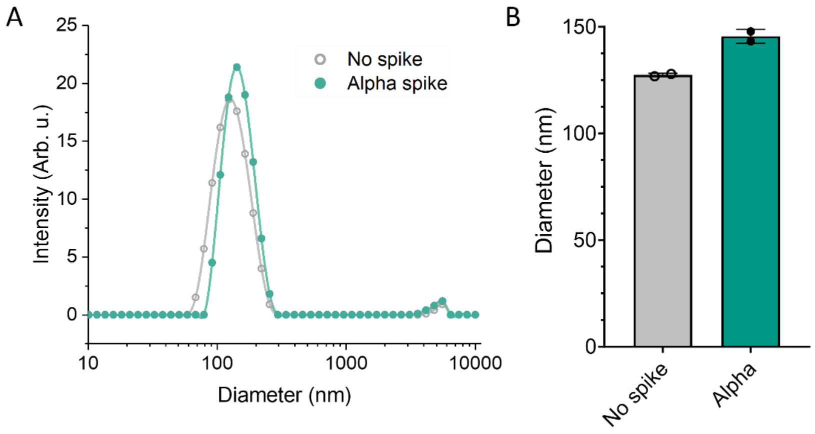

Figure S7 A. Size distribution of naked NTA-liposomes and liposomes decorated with soluble spike (Alpha variant) measured by dynamic light scattering (DLS). Solid lines are the cubic spline interpolation of the experimental data (dots) **B.** Mean diameter of naked and spike-decorated liposomes calculated from DLS measurements.

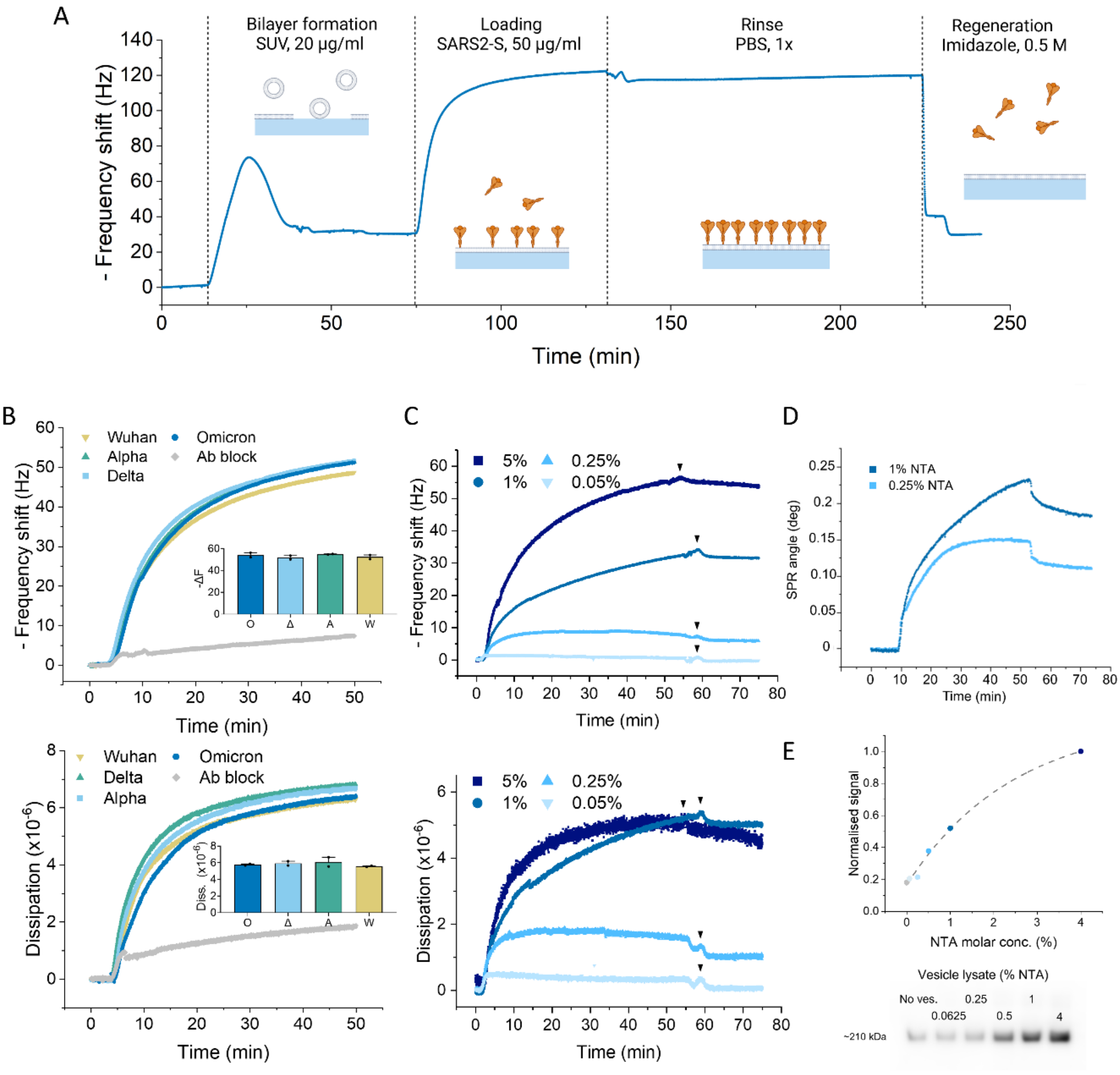

Figure S8 A. QCM-D measurement of spike attachment onto an NTA-presenting bilayer (1% molar percentage of NTA lipids). SUVs (99% POPC, 1% DGS-NTA, 20 mM $NiCl_2$) are injected over a SiO_2-coated sensor to form an SLB. Spike trimers (Omicron) are injected and incubated without flow until saturation. The formed bond is stable as no significant release is observed after rinsing in PBS for >1h. The almost complete removal of the protein by imidazole (0.5 M) indicates the specificity of the bond. **B.** QCM-D measurements, frequency shift (*Top*) and dissipation (*Bottom*), of the attachment of spike from all variants onto an NTA-bilayer (NTA-lipid molar fraction: 1%). Ab block (grey) indicates that preincubation of spike in 5-times excess of anti-His-tag antibody, which targets the His-tag on the spike protein prevents binding to the bilayer. Inset: Equilibrium values obtained after rinsing with buffer in two independent measurements. The values differ less than 5% indicating that spike interacts with NTA-presenting membranes similarly with all variant types. Each dot indicates an independent repeat, and the error bars indicate the standard error of the mean. **C.** QCM-D measurement, frequency shift (*Top*) and dissipation (*Bottom*), of the binding of soluble His-tagged spike on an NTA-presenting SLB, showing the dependency of spike binding to the concentration of NTA-conjugated lipids in the membrane. Molar fraction of NTA-lipids between 0.05% to 5%. Black triangles indicate the start of the rinsing in PBS. **D.** SPR measurement of spike attachment to NTA-presenting SLBs with a molar fraction of NTA-lipids of 0.25% and 1% used to estimate the spike density on the particles. **E.** *(Top)* Normalised spike content of the lysate from spike-decorated liposome with DGS-NTA molar concentration between 0 and 5%. The dashed grey line is the best exponential fit ($y = A + B\exp(-x/C)$) showing linearity up to 1%. *(Bottom)* Immunoblotting detection of spike in the lysates of spike-decorated liposomes used to generate the graph above.

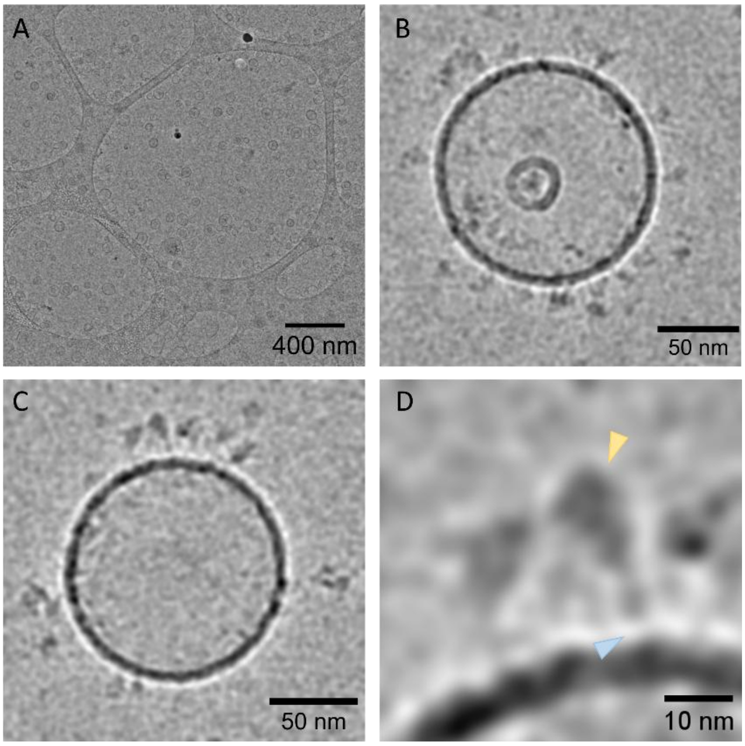

Figure S9 Cryo-EM images of spike-decorated liposomes (Alpha variant). **A.** Low magnification image showing the distribution of liposomes on the grid. **B-C.** Single liposomes displaying spike trimers on their surface. **D.** Detail of the structure of the spike trimer showing the correct orientation with the N-terminal domain exposed (yellow) and the C-terminus bound to the liposome surface (blue).

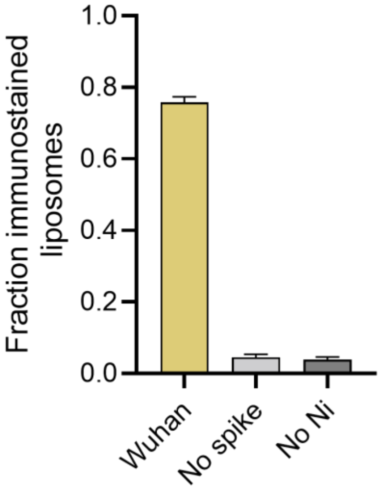

Figure S10 Fraction of spike-decorated liposomes presenting spike on their surface as measured by immunostaining with anti-SARS-CoV-2 spike antibody. The error bars are the standard deviation calculated from 12 images. The control “no spike” indicates that no spike was added when functionalizing the liposomes and “no Ni” indicates the liposomes were not preincubated in 20 mM of $NiCl_2$ before exposure to spike.

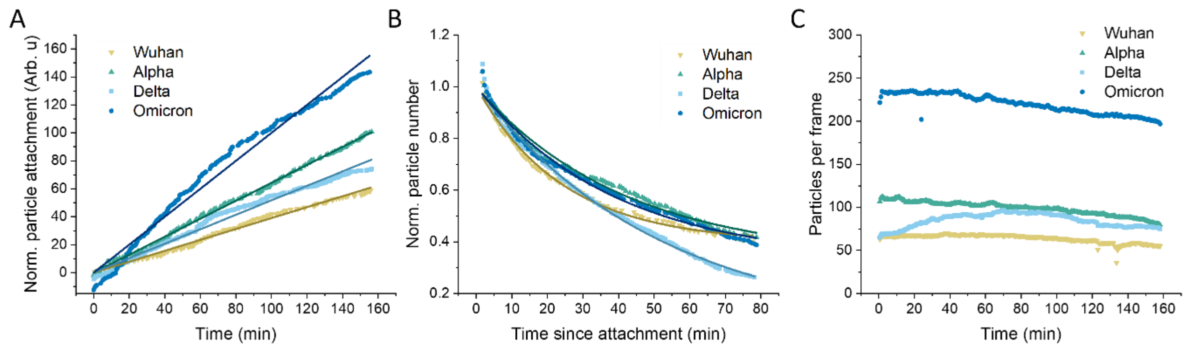

Figure S11 A. Example graph of the cumulative attachment of spike-decorated vesicles from different variants with the linear fit used to extract the association rate (k_{on}). The cumulative attachment is expected to be linear at kinetic equilibrium, as the rate of attachment is proportional to the association rate constant of the two species and the particle concentration in solution, which remain constant at equilibrium. The graph is rescaled so that the linear fit of Omicron has a slope of 1 min^{-1}. This is the same normalization used in Fig.3B,E and 4A in the main text. **B.** Example graph of the number of particles attached to the surface vs. the time elapsed since their attachment (τ). The solid line indicates the single exponential fit with offset used to calculate the dissociation rate (k_{off}). Curves are rescaled so the exponential fit is 1 at τ =0. The total number of particles considered in the analysis is: Omicron: 497, Delta: 886, Alpha: 425, Wuhan: 374. **C.** Example graph showing that the surface coverage, i.e. number of particles detected per frame, remain constant during the experiment and thus confirming kinetic equilibrium.

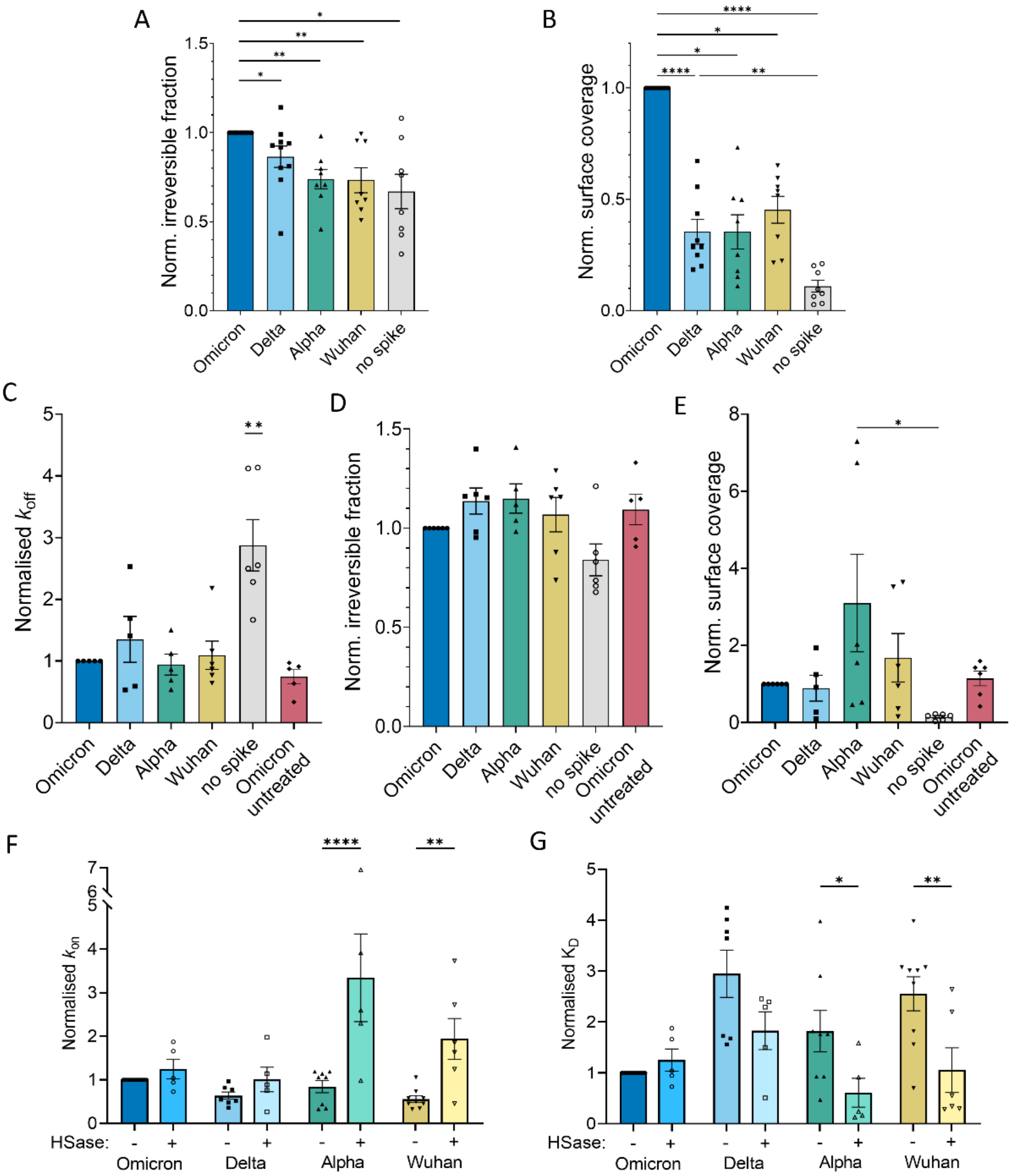

Figure S12 A-B. Fraction of spike-decorated liposomes forming a bond with a lifetime longer than 2500 s (Irreversible fraction) (A) and surface coverage (B) on Calu-3-derived nSLBs. **C-E.** Dissociation rate constant k_{off} (C), irreversible fraction (D), and surface coverage (E) of spike-decorated liposomes from VOCs on nSLBs treated with heparinase I and III. **F-G.** Association rate constant (F) and dissociation constant (G) for each variant, with or without enzymatic removal of HS (HSase). Statistical significance is calculated only within each variant. Statistical significance was calculated using one-way ANOVA test. *: p<0.05, **: p<0.01, ***: p<0.001. ****: p<0.0001. In B, ** indicates the lowest significance between the difference in the negative control and all the other conditions tested.

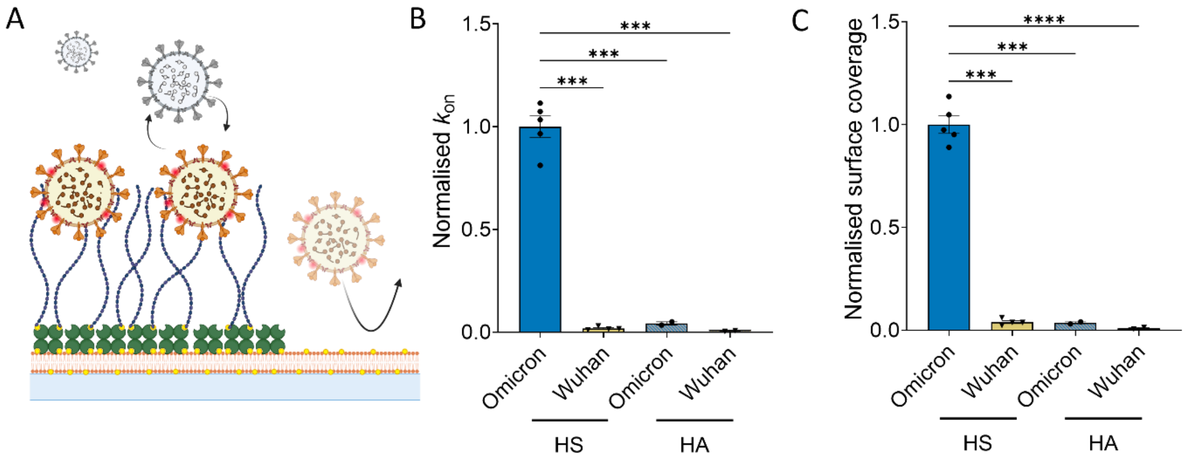

Figure S13 A. Schematic of the immobilization of biotinylated HS to a biotin-presenting supported lipid bilayer via a streptavidin bridge. This allows the direct observation of the binding of fluorescently labelled UV-inactivated SARS-CoV-2 virions to HS via TIRFM. **B.** Association rate constant for UV-inactivated Omicron and Wuhan virions on HS and HA. **C.** Average number of particles bound to the HS surface at equilibrium. P-values were determined by Brown-Forsythe and Welch ANOVA test: ***: $p<0.001$, ****: $p<0.0001$.

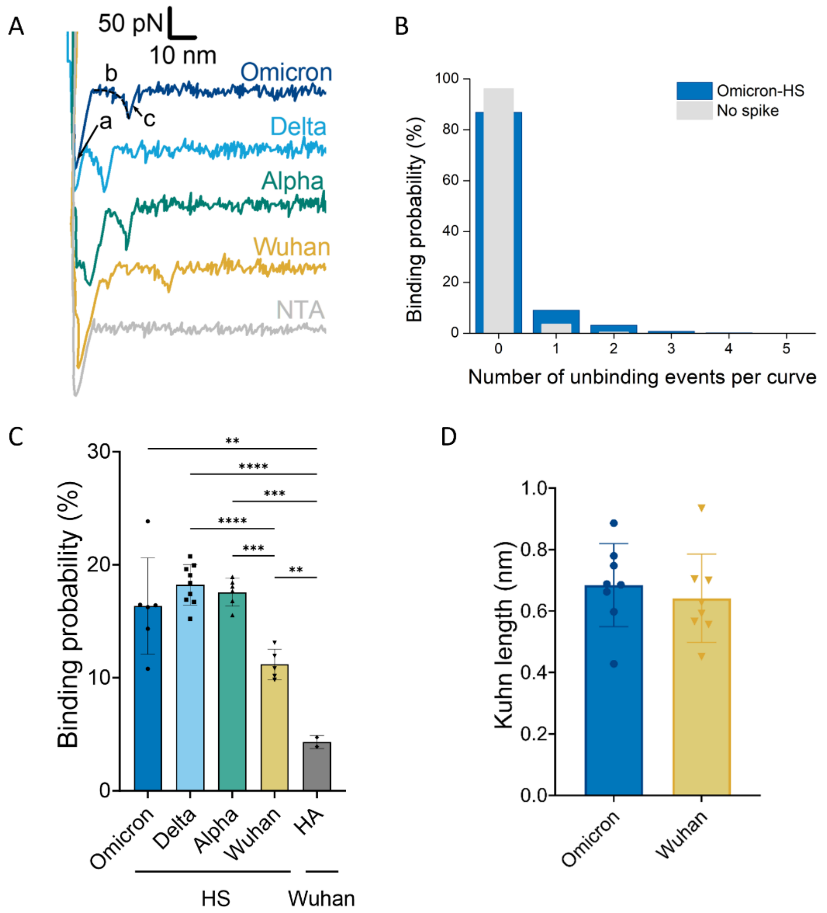

Figure S14 A. Exemplary force-distance curves to display specific unbinding events for all VOCs and AFM tip without spike (NTA). (a) Unspecific peak, (b) PEG stretching, and (c) spike-HS bond rupture. **B.** Binding probability for Omicron-HS (blue) and NTA-HS (light grey) in AFM-based SMFS experiments. **C.** Binding probability in percentage fraction at retract velocity of 1 µm/s and surface dwell time >0.3 s from at least 2 independent experiments to identify the specificity of spike variants binding to HS**. D.** Kuhn lengths of the PEG chain used to immobilize spike variants on the AFM tips for SMFS experiments. Average values of 0.68 ± 0.13 (Omicron) and 0.64 ± 0.14 nm (Wuhan).

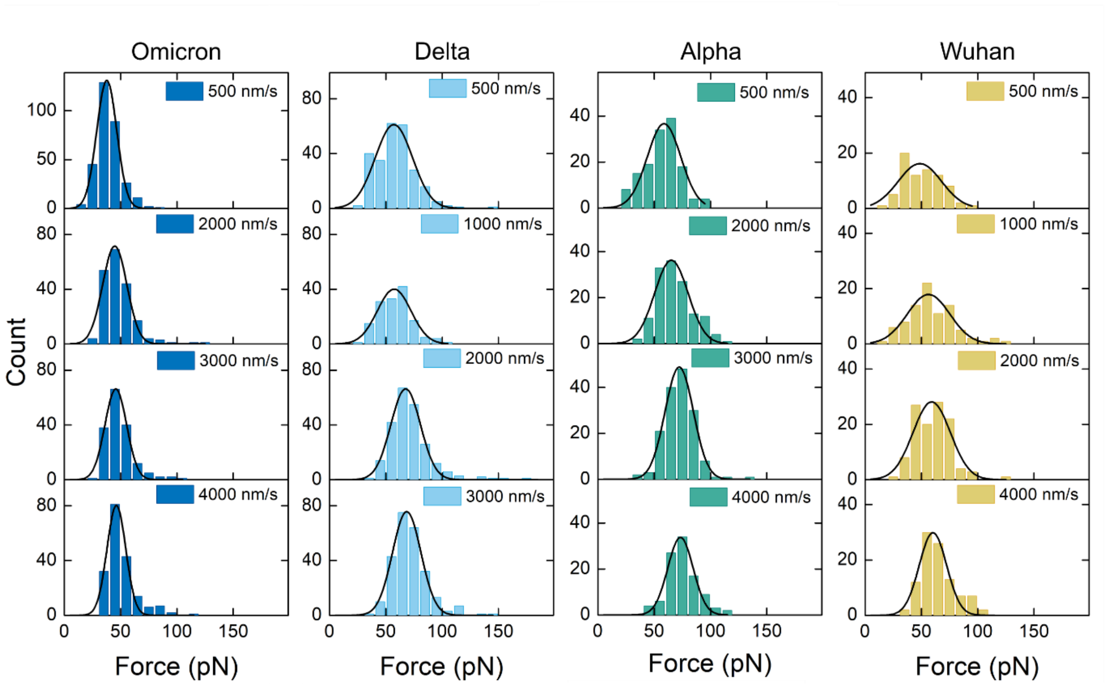

Figure S15 Rupture force distribution for spike-HS interactions for four variants as obtained by AFM-based SMFS.

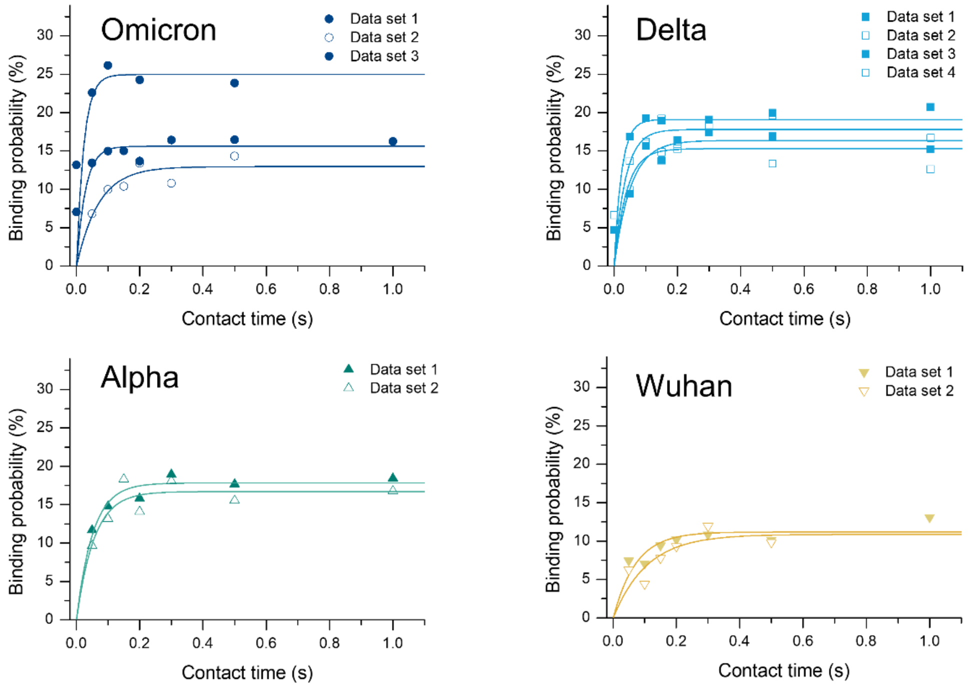

Figure S16 Binding probability data as a function of contact time for all four variants used to determine interaction time (τ) by fitting the binding probability with a mono-exponential function (solid lines).

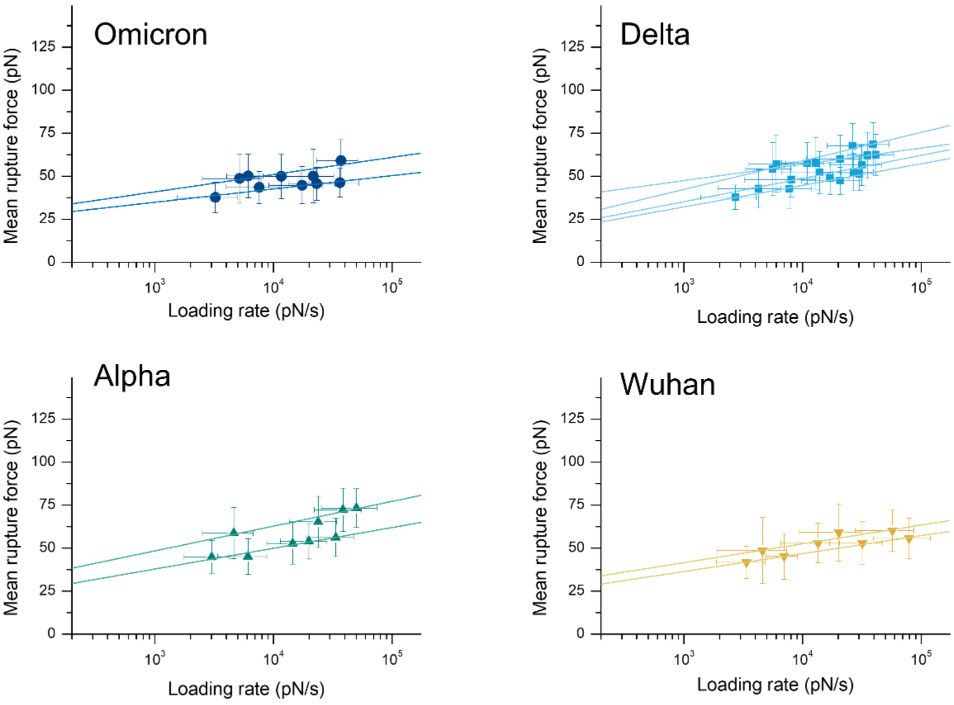

Figure S17 DFS data sets for all four variants with error bars (sigma from Gaussian fit of force histograms) to determine k_{off} and x_{β} by fitting DFS according to the Bell-Evans model (solid lines).

Supporting tables

	Omicron	Delta	Alpha	Wuhan	No spike
k_m (Arb.u.)	1.61 (0.34)	0.94 (0.11)	1.30 (0.49)	1.51 (0.50)	0.28 (0.08)
k_{off} (s^{-1}) ($x10^{-4}$)	4.14 (0.62)	8.45 (2.33)	4.75 (0.78)	4.49 (0.71)	27.0 (8.43)
Irreversible fraction (%)	53.8 (4.3)	42.9 (4.7)	47.7 (4.2)	48.1 (4.7)	33.0 (4.4)

Table S1 Mean and standard error of the mean, in brackets, of the kinetic parameters of the multivalent interaction between spike-decorated liposomes and Calu-3 derived nSLBs, prior to the normalization to the Omicron sample used in Fig.3B-D and Fig. S12A,B.

	Omicron	Delta	Alpha	Wuhan	No spike	Omicron (no hep.se)
k_m (Arb.u.)	2.78 (0.44)	1.98 (0.32)	7.79 (2.06)	4.61 (0.14)	0.67 (0.14)	2.59 (0.55)
k_{off} (s^{-1}) ($x10^{-4}$)	8.30 (1.78)	13.3 (6.12)	8.49 (2.53)	10.4 (4.43)	25.4 (3.87)	5.44 (0.68)
Irr. fraction (%)	50.1 (2.9)	54.3 (1.7)	55.3 (3.3)	51.3 (3.4)	41.3 (4.4)	52.6 (3.2)

Table S2 Mean and standard error of the mean, in brackets, of the kinetic parameters of the multivalent interaction between spike-decorated liposomes and heparinase (hep.se) treated Calu-3 derived nSLBs, prior to the normalization to the Omicron sample used in Fig.3E-H and Fig. S12C-G.

	Omicron	Delta	Alpha	Wuhan	HA
k_m (Arb.u.)	4.92 (1.30)	0.50 (0.18)	0.27 (0.05)	0.19 (0.05)	0.08 (0.04)
Surface coverage (particle/frame)	2987 (777)	283 (73)	100 (19)	95 (22)	33 (13)

Table S3 Mean and standard error of the mean, in brackets, of the kinetic parameters of the multivalent interaction between spike-decorated liposomes and SLB-immobilized heparan sulfate prior to the normalization to Omicron used in Fig.4A,B.

Supporting references

1. Peerboom, N. *et al.* Binding Kinetics and Lateral Mobility of HSV-1 on End-Grafted Sulfated Glycosaminoglycans. *Biophys. J.* **113**, 1223–1234 (2017).

2. Liu, L. *et al.* Recruitment of apolipoprotein E facilitates Herpes simplex virus 1 release. *bioRxiv* 2023.02.10.526562 (2023) doi:10.1101/2023.02.10.526562.

3. Stocks, B. B., Thibeault, M. P., Schrag, J. D. & Melanson, J. E. Characterization of a SARS-CoV-2 spike protein reference material. *Anal. Bioanal. Chem.* **414**, 3561–3569 (2022).

4. Wang, H. *et al.* Glutaraldehyde Modified Mica: A New Surface for Atomic Force Microscopy of Chromatin. *Biophys. J.* **83**, 3619–3625 (2002).

5. Ferrand-Drake del Castillo, G., Emilsson, G. & Dahlin, A. Quantitative Analysis of Thickness and pH Actuation of Weak Polyelectrolyte Brushes. *J. Phys. Chem. C* **122**, 27516–27527 (2018).

6. Ferrand-Drake del Castillo, G., Hailes, R. L. N. & Dahlin, A. Large Changes in Protonation of Weak Polyelectrolyte Brushes with Salt Concentration—Implications for Protein Immobilization. *J. Phys. Chem. Lett.* **11**, 5212–5218 (2020).

7. Andersson, J. *et al.* Control of Polymer Brush Morphology, Rheology, and Protein Repulsion by Hydrogen Bond Complexation. *Langmuir* **37**, 4943–4952 (2021).

8. Andersson, J. *et al.* Polymer Brushes on Silica Nanostructures Prepared by Aminopropylsilatrane Click Chemistry: Superior Antifouling and Biofunctionality. *ACS Appl. Mater. Interfaces* **15**, 10228–10239 (2023).

9. Emilsson, G. *et al.* Strongly Stretched Protein Resistant Poly(ethylene glycol) Brushes Prepared by Grafting-To. *ACS Appl. Mater. Interfaces* **7**, 7505–7515 (2015).

10. Dahlin, A. B. *et al.* Plasmonic Nanopores in Metal-Insulator-Metal Films. *Adv. Opt. Mater.* **2**, 556–564 (2014).

11. Ke, Z. *et al.* Structures and distributions of SARS-CoV-2 spike proteins on intact virions. *Nature* **588**, 498–502 (2020).

12. Sender, R. *et al.* The total number and mass of SARS-CoV-2 virions. *Proc. Natl. Acad. Sci.* **118**, (2021).

13. Sauerbrey, G. Verwendung von Schwingquarzen zur Wägung dünner Schichten und zur Mikrowägung. *Zeitschrift für Phys.* **155**, 206–222 (1959).

14. Pace, H. *et al.* Preserved Transmembrane Protein Mobility in Polymer-Supported Lipid Bilayers

Derived from Cell Membranes. *Anal. Chem.* **87**, 9194–9203 (2015).

15. Thorsteinsson, K., Olsén, E., Schmidt, E., Pace, H. & Bally, M. FRET-Based Assay for the Quantification of Extracellular Vesicles and Other Vesicles of Complex Composition. *Anal. Chem.* **92**, 15336–15343 (2020).

16. Jönsson, P., Jonsson, M. P., Tegenfeldt, J. O. & Höök, F. A Method Improving the Accuracy of Fluorescence Recovery after Photobleaching Analysis. *Biophys. J.* **95**, 5334–5348 (2008).
